# Outcomes in the Treatment of Subretinal Macular Hemorrhage Secondary to Age-Related Macular Degeneration: A Systematic Review

**DOI:** 10.3390/jcm13020367

**Published:** 2024-01-09

**Authors:** Filippo Confalonieri, Vanessa Ferraro, Gianmaria Barone, Alessandra Di Maria, Beáta Éva Petrovski, Josè Luis Vallejo Garcia, Alessandro Randazzo, Paolo Vinciguerra, Xhevat Lumi, Goran Petrovski

**Affiliations:** 1 Department of Ophthalmology, IRCCS Humanitas Research Hospital, Rozzano, 20089 Milan, Italy; vanessa.ferraro@humanitas.it (V.F.); gianmaria.barone@humanitas.it (G.B.); alessandra.di_maria@humanitas.it (A.D.M.); jose_luis.vallejo_garcia@humanitas.it (J.L.V.G.); paolo.vinciguerra@humanitas.it (P.V.); 2 Department of Biomedical Sciences, Humanitas University, Pieve Emanuele, 20090 Milan, Italy; 3 Center for Eye Research and Innovative Diagnostics, Department of Ophthalmology, Institute for Clinical Medicine, University of Oslo, Kirkeveien 166, 0450 Oslo, Norway; beata.petrovski@odont.uio.no (B.É.P.); xhevat.lumi@kclj.si (X.L.); 4 Department of Ophthalmology, Oslo University Hospital, Kirkeveien 166, 0450 Oslo, Norway; 5 Eye Hospital, University Medical Centre Ljubljana, Zaloška Cesta 2, 1000 Ljubljana, Slovenia; 6 Department of Ophthalmology, University of Split School of Medicine and University Hospital Centre, 21000 Split, Croatia; 7 UKLONetwork, University St. Kliment Ohridski-Bitola, 7000 Bitola, North Macedonia

**Keywords:** subretinal macular hemorrhage (SRMH), age-related macular degeneration (AMD), vitreoretinal surgery, recombinant tissue plasminogen activator (rt-PA)

## Abstract

**Background**: Subretinal macular hemorrhage (SRMH) secondary to age-related macular degeneration (AMD) is a relatively rare condition in ophthalmology characterized by blood collection between the neurosensory retina and the retinal pigment epithelium (RPE). Without prompt treatment, visual prognosis is poor. A plethora of treatment approaches have been tried over the past years ranging from intravitreal anti-vascular endothelial growth factor (anti-VEGF) monotherapy to direct subretinal surgery, with no conclusive superiority of one over the other. **Materials and Methods**: We conducted a systematic review of the outcomes and treatment modalities of SRMH from inception to 14 June 2022, following the Preferred Reporting Items for Systematic Reviews and Meta-Analyses guidelines (PRISMA). The level of evidence was assessed for all included articles according to the quality of evidence according to the Grading of Recommendations Assessment, Development and Evaluation (GRADE) system. **Results**: A total of 2745 articles were initially extracted, out of which 1654 articles were obtained after duplicates were removed and their abstracts screened. A total of 155 articles were included for full-text review. Finally, 81 articles remained that fulfilled the inclusion criteria. **Conclusions**: Even though there are solid results supporting a variety of treatments for SRMH, the best treatment modality has still not been conclusively demonstrated and further research is needed.

## 1. Introduction

Retinal hemorrhage is among the most common clinical signs in retinal disease and consists of a spectrum of blood collection differing in location, size, distribution, and etiology [1]. Fovea-involving subretinal macular hemorrhage (SRMH) is a sight-threatening condition defined as blood collection between the neurosensory retina and the retinal pigment epithelium (RPE) [2]. SRMH can be caused by a plethora of eye disorders, including neovascular age-related macular degeneration (n-AMD) and its variants such as polypoid choroidal vasculopathy (PCV), but also pathologic myopia, ruptured retinal artery macroaneurysms, presumed ocular histoplasmosis syndrome, and trauma [3,4,5,6,7]. SRMH can cause irreversible damage to the photoreceptors; if left untreated, a blood clot under the retina usually turns into a scar, causing permanent loss of central vision [8,9,10].

AMD is the leading cause of legal blindness in the industrialized world [11]. The real incidence of SRMH among patients with n-AMD is unknown [12], even though n-AMD has long been known to be a risk factor for submacular bleeding [8,13].

SRMHs larger than one disc diameter (DD) across in size have been reported in 24 people per million per year, according to a population-based study conducted in two UK centers, while SRMHs larger than two DDs have been reported in only 5.4 people per million per year in a study by a Scottish Ophthalmic Surveillance Unit (SOSU) [14,15]. Nevertheless, the population in many countries is ageing and the disease prevalence for AMD, and therefore SRMHs, is supposed to increase significantly in the coming years [16].

SRMH generally results in a severe and irreversible loss of vision, ranging from 6/30 to light perception, if left untreated [17]. Moreover, only 11% of the eyes in the control group of a submacular surgery study achieve a final best-corrected visual acuity (BCVA) higher than 6/60 [10]. The functional outcome may also be influenced by the duration and size of SRMH, as well as the etiology and location of the bleeding source. Persistent SRMH damages the photoreceptors through three main mechanisms: iron-related toxicity, impairment of diffusion of oxygen and nutrition, and mechanical damage due to clot contraction [18,19,20,21,22,23]. The natural history of SRMH typically leads to a central scotoma with a fibrotic macular scar (38%), atrophy (25%), or RPE rupture (22%) [17].

A variety of approaches have been employed in the treatment of SRMH, and even though ample literature exists, this is dispersive and predominantly made up of small, single-center outcome reports that do not encompass all the therapeutic techniques that have been described.

The purpose of this systematic review is to analyze and summarize the current therapeutic approaches in the management of SRMH while evaluating the level and quality of the research included.

## 2. Materials and Methods

A systematic review was conducted and reported according to the Preferred Reporting Items for Systematic Reviews and Meta-Analyses (PRISMA) guidelines [24]. The review protocol was not recorded in the study design, but a registration number will be available for consultation. The methodology used consisted of a systematic search of all available articles exploring the treatment modalities of SRMH secondary to n-AMD. To identify all relevant published articles, we performed a systematic literature search including papers published from inception until 14 June 2022. These were searched in Ovid Medline, Embase, Cochrane Register of Controlled Trials, and Cochrane Database of Systematic Reviews using controlled vocabulary and text words expressing (subretinal OR submacular) AND (hemorrhage OR haemorrhage OR bleeding). The search was not restricted by publication type, study design, or date of publication. The search was restricted by the English language. The complete search strategy is given in Appendix A.

Subsequently, the reference lists of all identified articles were examined manually to identify any potential study not selected by the electronic searches. After the preparation of the list of all electronic data, a reviewer (FC) examined the titles and abstracts and identified relevant articles. All the studies analyzing outcomes of the available treatment modalities of SRMH in n-AMD were considered as satisfactory for the inclusion criteria. Exclusion criteria were review studies, pilot studies, letters to the editor, case series with ≤12 eyes, case reports, photo essays, and studies written in languages other than English. Moreover, studies performed on animal eyes, cadaveric eyes, and pediatric patients were excluded as well. Exclusion criteria also included studies that were not specifically powered to detect a correlation between the treatment modality of either the anatomical or functional outcomes in SRMH treatment. SRMH secondary to diseases other than n-AMD was also excluded.

The same reviewer registered and selected the studies according to the inclusion and exclusion criteria by examining the full text of the articles. Any doubt was assessed by consensus with a third-party reviewer (GP), who was consulted when necessary. No further unpublished data were obtained from the corresponding authors of all selected articles, which were analyzed to assess the level of evidence according to the quality of evidence according to the Grading of Recommendations Assessment, Development and Evaluation (GRADE) system [25,26].

## 3. Results

A total of 2745 articles were initially extracted. Consequently, 1654 articles were obtained after the duplicates were removed and their abstracts were screened. Subsequently, 155 articles were included for the full-text review and more in-depth evaluation of the inclusion/exclusion criteria. Finally, 81 articles remained that fulfilled all the inclusion criteria.

**Figure 1:**
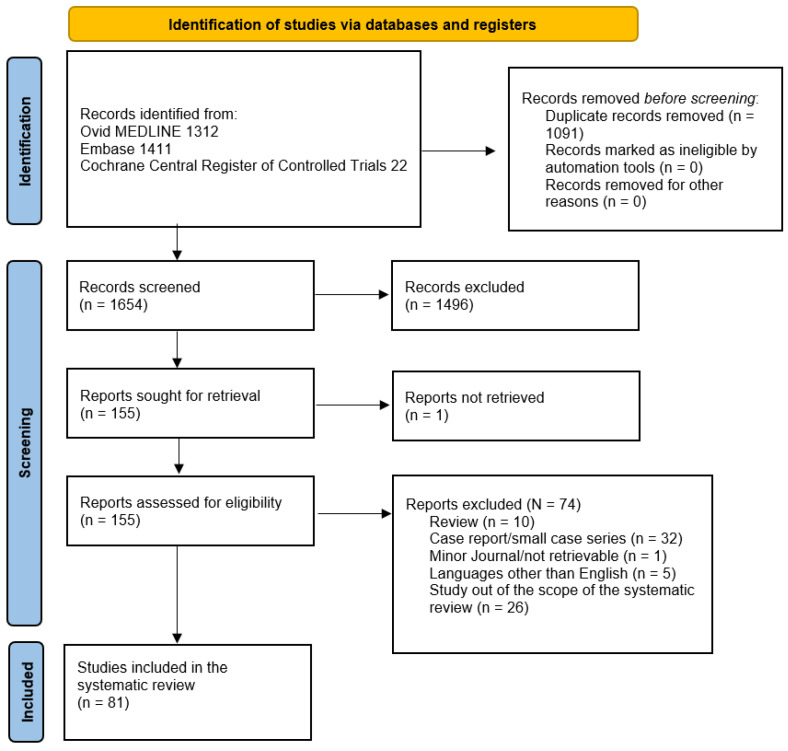
Flowchart of the literature search and selection according to Preferred Reporting Items for Systematic Reviews and Meta-Analyses guidelines (PRISMA) [24].

Figure 1 summarizes the research approach applied here in a flowchart.

The determining reasons for inclusion or exclusion of the full-text reviewed articles are summarized in Appendix B. Furthermore, Appendix C summarizes all the studies extracted from the systematic literature search, with the relevant descriptive information.

In order to summarize the large amount of information derived from the systematic search, Table 1 was created to report on the studies that are prospective or randomized controlled trials (RCTs). These are in fact the most valuable studies, and they are the main source of evidence.

In Table 2, the studies were grouped according to the type of intervention that was applied, which were anti-vascular endothelial growth factor (anti-VEGF) only, intravitreal recombinant tissue plasminogen activator (rt-PA), and/or subretinal rt-PA. 

In Table 3, a summary of the studies is provided according to the hemorrhage onset, as the timing of intervention seems to be crucial for the outcome of SRMH patients [55].

Finally, Table 4 groups all the included studies on the basis of SRMH size in an attempt to simplify the understanding for prognostic purposes. 

## 4. Discussion

SRMH poses a formidable challenge in the realm of retinal pathology, given its potential to cause irreversible damage to central vision. Despite its clinical significance, the optimal treatment strategy for SRMH remains to be an ongoing debate, largely due to the scarcity of comprehensive prospective studies and the consequent absence of a widely accepted best practice.

This systematic review critically assessed the existing literature on SRMH treatment modalities, focusing on prospective studies to elucidate the current landscape of therapeutic interventions. The number of prospective trials specifically targeting SRMH is limited, thus underscoring the need for further robust investigations to guide evidence-based decision-making. Within the sparse collection of prospective studies, various treatment options have been explored, ranging from conservative observation to surgical interventions. Notably, only retrospective studies by Ueda-Arakawa et al. [103] and Maggio et al. [47] have explored the merits of a watchful waiting approach, positing its suitability for cases marked by minimal visual impairment and self-resolving hemorrhages. Nevertheless, due to the relatively small sample sizes and inherent variability in hemorrhage characteristics, these studies have not been able to definitively establish the superiority of observation over the active therapeutic interventions.

Pneumatic displacement, an innovative approach, has gained attention for its potential to physically displace subretinal hemorrhage away from the macula. The prospective investigations by Gopalakrishan et al. [28] and De Jong et al. [31] unveiled encouraging results, suggesting improved visual outcomes. However, the limited number of patients and the absence of long-term follow-up data cast a shadow over the sustainability of these positive findings.

Anti-VEGF agents, with their established efficacy in various retinal pathologies, have been examined as a potential treatment modality for SRMH. Iacono et al. [29] conducted prospective studies probing the impact of anti-VEGF injections on neovascularization and inflammation associated with SRMH. Despite the promise showcased in this study, the lack of consensus in the treatment regimens and the modest sample sizes hinder the establishment of a definitive therapeutic role for anti-VEGF agents.

Surgical interventions, specifically vitrectomy with or without rt-PA injection, have been a subject of exploration through prospective studies by Wei et al. [30], Mozafarieh et al. [27], Kadonosono et al. [86], Kimura et al. [69], and De Jong et al. [31]. These studies have shed light on the potential benefits of surgical intervention, particularly in cases of larger and dense or recurrent hemorrhage. However, the invasiveness of the procedure, coupled with concerns regarding complications, necessitates judicious patient selection and cautious consideration of risks and benefits.

Photodynamic therapy (PDT), a modality with established efficacy in other retinal conditions, has also found its way into the discourse surrounding SRMH treatment. Notable retrospective studies by Lin et al. [44] have ventured into investigating PDT’s potential role in addressing neovascularization in SRMH. Nevertheless, the existing body of evidence is marked by its infancy and a lack of consistent findings, impeding the establishment of PDT as a definitive treatment avenue.

Our work sums up all the available treatment modalities of SRMH. As the management of this condition is a complex and challenging task, several treatment modalities have been developed to address it, each with its own set of advantages and limitations. We will further highlight the various treatment modalities for SRMH below. 

Intravitreal Anti-VEGF Therapy: Intravitreal injection of anti-VEGF agents, such as ranibizumab and bevacizumab, has gained popularity in recent years. These drugs can resolve SRMH by inhibiting abnormal blood vessel growth and leakage in conditions like CNV. The advantages of this approach include its minimal invasiveness and relatively rapid resolution of the hemorrhage. However, it may not be effective in all cases, and multiple injections over an extended period of time may be required. The long-term safety profile of these agents also warrants the ongoing and further research. 

Pneumatic displacement involves the injection of expansile gases, such as sulfur hexafluoride or perfluoropropane, into the vitreous cavity. This gas displaces the SRMH, moving it away from the macula, allowing for improved vision. Pneumatic displacement is less invasive than other surgical procedures and can be an effective treatment option. However, it may be associated with complications, such as subretinal blood displacement, which necessitates careful patient selection and follow-up.

Vitrectomy is a surgical intervention that involves the removal of vitreous gel from the eye. This procedure allows direct visualization and access to the subretinal space, enabling the removal of blood and other substances. Vitrectomy is effective in a wide range of cases, particularly when the hemorrhage is extensive, the fibrosis has occurred, or when other treatment modalities have failed. However, it is an invasive procedure with potential surgical risks, longer recovery times, and need for careful postoperative management. Potential complications from pneumatic displacement during vitrectomy can include vitreous or choroidal hemorrhage, hyphema, RPE tear and cataract formation, retinal detachment, increased intraocular pressure/glaucoma, full-thickness macular hole formation, and endophthalmitis (Appendix C).

Subretinal injection of rt-PA followed by the injection of an expansile gas, such as sulfur hexafluoride or perfluoropropane can facilitate the mechanical displacement of the SRMH and potentially improve visual outcomes. However, it is a surgical procedure and requires experienced surgical skills to minimize risks.

The choice of treatment for SRMH should be individualized, considering factors such as the extent and location of the hemorrhage; the patient’s overall health, including blood pressure and cardiac status, use of blood thinners, and INR level where applicable; the visual acuity goals; and the potential risks and benefits associated with each option. A multidisciplinary approach, involving ophthalmologists, vitreoretinal surgeons, and the patient, is often crucial in making the most informed decision.

**Figure 2:**
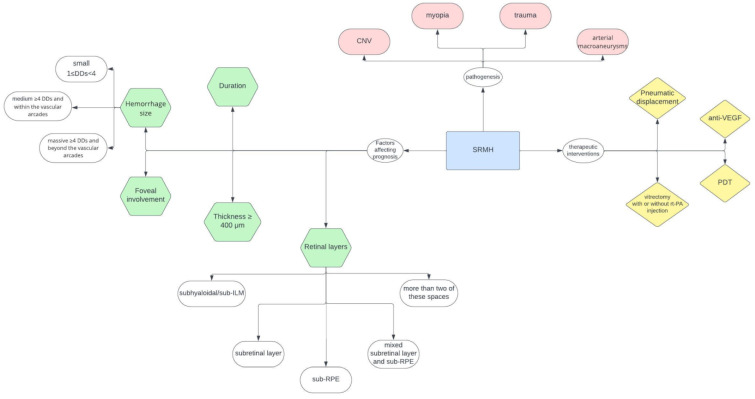
Flow chart on the clinical diagnostics, management, and treatment of patients with acute loss of vision due to suspected SRMH. Disc diameter (DD); photodynamic therapy (PDT).

A flow chart on the clinical diagnostics, management, and treatment of patients with acute loss of vision due to suspected SRMH is depicted in Figure 2.

As research continues to evolve, new treatment modalities and refinements to existing approaches may emerge, offering hope for improved outcomes and quality of life for individuals affected by SRMH. The optimal approach to managing this condition will depend on the specific characteristics and needs of each patient, and ongoing clinical trials and research will help shape the future of SRMH treatment.

## 5. Conclusions

In conclusion, the management of subretinal macular hemorrhage presents a complex and challenging clinical scenario. Various treatment modalities have been explored, each with its own set of pros and cons. The choice of treatment should be tailored to the individual patient, taking into consideration the specific characteristics of the hemorrhage, the patient’s overall health, and their visual acuity goals.

Intravitreal injection of anti-VEGF agents has emerged as a promising non-invasive option for some patients. Its advantages include rapid resolution of hemorrhage, minimal invasiveness, and a potential for improved visual outcomes. However, it may not be effective in all cases, especially in instances of massive hemorrhage or when fibrotic changes have already occurred. Additionally, the need for multiple injections and the long-term safety profile of these drugs require ongoing research.

Surgical interventions, such as pneumatic displacement and vitrectomy, offer the advantage of direct visualization and removal of the hemorrhage. These procedures can be effective in a wider range of cases and may yield significant improvements in vision. Nevertheless, they come with the risk of surgical complications, prolonged recovery periods, and potential long-term anatomical changes. The choice of surgery should be made carefully, considering the individual patient’s surgical risk profile and the likelihood of postoperative complications.

The use of subretinal tPA and gas injection, while it may achieve faster resolution compared to observation alone, it is still an invasive procedure like traditional vitrectomy. This technique is, however, not suitable for all cases and requires experienced surgical hands to minimize risks.

Ultimately, the decision on the most appropriate treatment modality for subretinal macular hemorrhage should be made through a multidisciplinary approach involving the ophthalmologist, the patient, and other healthcare providers. It is imperative to weigh the potential benefits against the risks and limitations of each approach while considering the patient’s individual circumstances and preferences. Ongoing research and clinical trials will continue to refine our understanding of these treatment modalities and potentially lead to further advancements in the management of this challenging condition. As we move forward, it is crucial that clinicians remain vigilant in their pursuit of improved therapies, with the goal of optimizing visual outcomes and enhancing the quality of life for patients with subretinal macular hemorrhage.

## Figures and Tables

**Table 1:** Prospective or RCT studies on subretinal bleeding.

References	Year	Study Design	Study Sample (Eyes)	Type of Surgery	Mean Size of the Bleeding	Outcome Final BCVA	Mean Days from Onset	Complications	GRADE ^1^
[27]	2006	Longitudinal PROSP	101	IVT PA	>1 DD	-	<4 weeks	None	Moderate
[28]	2007	PROSP, CONSEC, single-center, NComp, ITRV, case series	20	IVT C_3_F_8_ without rt-PA	N/A	Improved	Range from 1 to 30 days	4 VH	Moderate
[29]	2014	PROSP, ITRV, case series	23	IVT ranibizumab	Occult choroidal neovascularization with flat large submacular hemorrhage > 50% of the entire lesion	Improved	N/A	None	Very low
[30]	2015	PROSP, NRandom, NComp, case series	21	PPV + 360° retinotomy + silicon oil (Oxane 5700) tamponade	N/A	Improved	N/A	10 mild subretinal fibrosis	Moderate
[31]	2016	PROSP, NComp, ITRV, case series	24	Group A: PPV + gas + subretinal rt-PA; Group B: IVT rt-PA + gas	Group A: 11.1 DA (range 0.5–31.0); Group B: 9.7 DA (range 2.9–20.2)	Improved	Group A: 5 (range 1–11), Group B: 6 (range 1–14)	Group A: 3 increased IOP > 50 mmHg, 2 VH, 1 RD, 1 recurrence. Group B: 2 RD, 2 recurrences	Very low
[32]	2016	PROSP, ITRV, CONSEC, case series	20	IVT rt-PA + ranibizumab + gas without PPV	11.1 ± 8.7 DD (range: 2–31)	Improved	9.9 ± 9.8 days (range 2–30)	3 VH, 1 RD	Very low
[33]	2022	Extended study of previous PROSP study	64	IVT rt-PA + ranibizumab + gas	8 ± 6 (range, 2–27) disc diameters	Improved	7 ± 7 days (range 1–30)	46 recurrences	Low
[34]	2021	Secondary analyses of an RCT of image and clinical data	535	Randomly divided: monthly IVT ranibizumab, as-needed IVT ranibizumab, monthly IVT bevacizumab, or as-needed bevacizumab	89% were < 1 DD	Improved	N/A	1 RPE tear, 28 fibrosis, 10 atrophic scars, 6 geographic atrophies, 7 epiretinal membranes	Moderate

Legend: CONSEC: consecutive series; IOP: intraocular pressure; ITRV: interventional; IVT: intravitreal; NComp: non-comparative; NRand: non-randomized; PROSP: prospective; RD: retinal detachment; rt-PA: recombinant plasminogen activator; VH: vitreous hemorrhage. ^1^ Grading of Recommendations Assessment, Development and Evaluation (GRADE) system [25,26].

**Table 2:** Studies on subretinal bleeding according to treatment strategies: anti-VEGF only vs. IVT rt-PA vs. subretinal rt-PA.

References	Study Design	Study Sample (No. of Eyes)	Type of Surgery	Mean Size of the Bleeding	Outcome Final BCVA	Mean Days from Onset	GRADE ^1^
[35]	NRand, Retro, ITRV, COMPR, CONSEC	47	PPV + IVT rt-PA + SF_6_ (group A) vs. PPV + subretinal rt-PA + SF_6_ (group B)	N/A	No significant difference	6.6 days (group A), 5.9 days (group B)	Low
[36]	NRand, Retro, ITRV, COMPR, CONSEC	38	IVT rt-PA + SF_6_ (group A) vs. IVT bevacizumab + rt-PA + SF_6_ (group B)	>1 DD	Significantly higher in group B	1–31 days	Low
[37]	NRand, Retro, COMPR, case study	110	rt-PA injection w/o gas injection (group A1: 50 µg of rt-PA; A2: 100 µg; A3: 200 µg) and with gas injection (group B1: 50 µg of rt-PA; B2: 100 µg; B3: 200 µg)	12.5 (1–38) DD	Better in B1 and B2 groups	10.0 (0.5–180.0)	Low
[38]	NRand, Retro, ITRV, COMPR, CONSEC	45	rt-PA (50 µg⁄0.05 mL) + SF_6_ (group A); bevacizumab (1.25 mg⁄0.05 mL) + SF_6_ (group B). Thereafter, all patients received bevacizumab	1–5 DD	Better in group A	N/A	Low
[39]	Retro, single-center study	46	PPV + subretinal rt-PA (group 1); pneumatic displacement + IVT rt-PA + gas (group 2); PD + gas (group 3)	5.6 ± 3.4 DD	No significant difference	10	Low
[40]	Retro, COMPR, ITRV, case series	32	PD (SF_6_) + IVT bevacizumab (group A) vs. PD (SF_6_) alone (group B)	>2 DD	Significantly better in group A	<10 days	Low
[41]	Retro, NComp, ITRV, case series	46	IVT bevacizumab (group A: 1–4 DD), group B (4–9 DD), group C (>9 DD)	6 DD	Amongst groups, improvement of the BCVA in 57% (13/23), 53% (8/15), and 38% (3/8) of eyes, respectively.	11.5 ± 19 days (range: 1–45 days)	Low
[42]	Retro, COMPR, ITRV, case series	82	PD (SF_6_ or C_3_F_8_) + IVT anti-VEGF vs. anti-VEGF monotherapy	N/A	No significant difference; combination therapy group showed better BCVA at 1 month after initial treatment compared to monotherapy	11.4 ± 10.4 days in the combination therapy group; 13.8 ± 11.5 days in the monotherapy group	Low
[31]	Prospective, NComp, ITRV, case series	24	PPV + gas + subretinal rt-PA (Group A); intravitreal rt-PA + gas (Group B)	Group A: 11.1 DD (range 0.5–31.0); Group B: 9.7 DD (range 2.9–20.2)	No significant difference	Group A: 5 (range 1–11), Group B: 6 (range 1–14)	Very low
[43]	Retro, case series	39	PPV + subretinal rt-PA (Group A); PD + IVT rt-PA (Group B); PD without rt-PA (Group C)	Group A: 9.1 mm^2^; Group B: 8.1 mm^2^; Group C: 9.1 mm^2^	Improved significantly in both Groups A and B, but not C	Group A: 5 ± 4.6; Group B: 6 ± 4.2; Group C: 6 ± 2.2	Low
[44]	Retro, COMPR, ITRV, case series	20	Group A: subretinal rt-PA + PPV; Group B: or intravitreal rt-PA + gas to achieve PD. Additionally, combination treatment with either PDT or IVT of anti-VEGF was performed	17.8 ± 19.2 disc diameter (DD) compared (2.64 DD)	Combination treatment with PDT showed significant efficacy in the improvement of BCVA	14.3 ± 16.6 days	Very low
[45]	Retro	18	PD followed by IVT rt-PA if needed vs. PPV with subretinal rt-PA	N/A	≥lines improvement at 1 year was 46% and 18% in the groups, respectively; no significant difference	N/A	Very low
[46]	Retro	77	Group A: anti-VEGF monotherapy; Group B: PD + anti-VEGF; Group C: PPV + subretinal rt-PA + gas tamponade	Three groups according to dimensions: small-sized (optic disc diameter (ODD) ≥ 1 to < 4), medium-sized (ODD ≥ 4 within the temporal arcade), and large-sized (ODD ≥ 4, exceeding the temporal arcade)	Small-sized group: all treatments had gradual BCVA improvement; medium-sized group: PD and surgery were associated with better BCVA than anti-VEGF monotherapy; large-sized group: surgery showed a better visual improvement with a higher displacement rate than PD	14.3 ± 25.8	Low
[47]	Retro, NComp, ITRV, case series	96	IVT rt-PA + SF_6_ for guiding the selection of additional treatments (anti-VEGF, PDT, or submacular surgery) or observation (CNV)	≥3 DA involving the fovea	BCVA improved significantly	<14 days	Low
Asli [48]	Retro, case series	54	PPV + submacular rt-PA + 20% SF_6_ or 14% C_3_F_8_; PPV + submacular rt-PA + 20% SF_6_ or 14% C_3_F_8_ + anti-VEGF; PPV+ subretinal rt-PA without gas; IVT gas + rt-PA; PPV + subretinal rt-PA + drainage; IVT gas + IVT anti-VEGF	31.5 ± 26.5 (2.8–145.3) mm^2^	BCVA improved	13.7 ± 16.3 (1–95) days	Low
[49]	Retro	29	Group 1: IVT rt-PA + SF_6_ Group 2: PPV + subretinal rt-PA + SF_6_ with (2A) or without (2B) subretinal air	9.45 ± 2.34 DD (Group 1) and 9.72 ± 2.02 DD (Group 2)	BCVA improved; Group 2: adding subretinal air gave no statistically significant difference in outcome	N/A	Very low
[50]	Retro	30	13 eyes PD, 22 eyes IVT anti-VEGF, 4 eyes PPV	17.0 ± 4.8 disc areas	BCVA improved	N/A	Low
[51]	Retro, CONSEC, case series	31	PPV + subretinal rt-PA + air displacement with or without IVT bevacizumab, respectively	11.78 ± 3.04 mm^2^ and 14.75 ± 3.98 mm^2^, respectively	BCVA improved significantly	3.3 ± 1.6 and 3.4 ± 1.5, respectively	Low
[52]	Retro	54	PPV + subretinal rt-PA + PD vs. anti-VEGF monotherapy	In rt-PA group: 5.0 DD; in anti-VEGF group: 4.2 DD	BCVA improved significantly for rt-PA group	5 days (range: 1–13)	Low
[53]	Retro, COMPR, ITRV, case series	25	Group A: PPV + subretinal rt-PA + gas; Group B: IVT rt-PA + gas	4.604 ± 2079 µm	BCVA improved significantly but did not differ between the 2 groups	8.2 ± 7.3 days	Very low
[34]	Secondary analyses of an RCT of image and clinical data	535	Randomly divided: monthly IVT ranibizumab, as-needed IVT ranibizumab, monthly IVT bevacizumab or as-needed bevacizumab	89% were <1 DD	BCVA improved	N/A	Moderate
[54]	Retro	107	Group A: IVT rt-PA + gas; Group B: PPV	767 µm in Group A; 962.5 µm in Group B	Better improvement in the rt-PA + gas group	N/A	Low

Legend: COMPR: comparative; CONSEC: consecutive series; DD: disc diameter; ITRV: interventional; IVT: intravitreal; NComp: non-comparative; NRand: non-randomized; PD: pneumatic displacement; RCT: randomized controlled trial; Retro: retrospective. ^1^ Grading of Recommendations Assessment, Development and Evaluation (GRADE) system [25,26].

**Table 3:** Summary of the mean days from onset: less or more than 2 weeks from symptoms’ onset in subretinal bleeding with references.

Treatment < 14 Days from Onset (Mean)	Treatment More than 14 Days from Onset (Mean)	Treatment Both before and after 14 Days from Onset or Not Specified/Not Clear
De Jong et al. [31], Kitagawa et al. [32], Kitagawa et al. [33], Hillenkamp et al. [35], Tsymanava et al. [37], Rishi et al. [39], Kitahashi et al. [40], Dimopoulus et al. [41], Shin et al. [42], Fassbender et al. [43], Maggio et al. [47], Asli Kirmaci Kabakci et al. [48], Rickmann et al. [51], Sniatecki et al. [52], Tranos et al. [53], Ratanasukon et al. [56], Singh et al. [57], Yang et al. [58], Stifter et al. [59], Arias et al. [60], Cakir et al. [61], Kung et al. [62], Sandhu et al. [63], Treumer et al. [64], Cho et al. [65], Jain et al. [66], Moisseiev et al. [67], Kim et al. [68], Kimura et al. [69], González-López et al. [70], Lee et al. [71], Waizel et al. [72], Gok et al. [73], Waizel et al. [74], Bardak et al. [75], Sharma et al. [76], Karamitsos et al. [77], Lee et al. [78], Ali Said et al. [79], Avci et al. [80], Iannetta et al. [81], Kawakami et al. [82], Pierre et al. [13], Fukuda et al. [83]	Lin et al. [44], Jeong et al. [46], Juncal et al. [84], Olivier et al. [85], Kadonosono et al. [84,86], Kim et al. [87], Caporossi et al. [88], Ura et al. [89]	Mozaffarieh et al. [27], Gopalakrishan et al. [28], Mehta et al. [34], Guthoff et al. [36], Mayer et al. [38], Yang et al. [58], Iacono et al. [29], Wei et al. [30], Bell et al. [45], Kishikova et al. [49], Matsuo et al. [50], Tiosano et al. [54], Ura et al. [89], Thompson et al. [90], Ron et al. [91], Meyer et al. [92], Fang et al. [93], Fine et al. [94], Mizutani et al. [95], Han et al. [96], Shienbaum et al. [97], Chang et al. [98], Kimura et al. [99], Plemel et al. [100], Helaiwa et al. [101], Wilkins et al. [102]

**Table 4:** Studies on subretinal bleeding according to the size.

References	Mean Size of the Bleeding (Disc Diameter (DD))
Mehta et al. [34], Tiosano et al. [54]	<1 DD
Mozaffarieh et al. [27], Guthoff et al. [36], Fassbender et al. [43], Asli Kimarci Kabakci et al. [48], Rickmann et al. [51], Arias et al. [60], Kung et al. [62], Jain et al. [66], Meyer et al. [92], Shienbaum et al. [97], Ura et al. [89]	1–3 DD
Iacono et al. [29], Rishi et al. [39], Dimopoulos et al. [41], Ratanasukon et al. [56], Kung et al. [62], Sandhu et al. [63], Treumer et al. [64], Kimura et al. [69], Avci et al. [80], Maggio et al. [47], Iannetta et al. [81], Pierre et al. [13], Thompson et al. [90], Ron et al. [91], Stifter et al. [59]	>3 DD
Iacono et al. [29], De Jong et al. [31], Kitagawa et al. [33], Mayer et al. [38], Kitahashi et al. [40], Jeong et al. [46], Kishikova et al. [49], Sniatecki et al. [52], Tranos et al. [53], Kimura et al. [69], Lee et al. [71], Gok et al. [73], Sharma et al. [76], Karamitsos et al. [77], Lee et al. [78], Kawakami et al. [82], Kim et al. [87], Juncal et al. [84], Helaiwa et al. [101], Ueda-arakawa et al. [103], Kim et al. [104]	<12 DD
Mozaffarieh et al. [27], De Jong et al. [31], Kitagawa et al. [32], Tsymanava et al. [37], Lin et al. [44], Matsuo et al. [50], Rickmann et al. [51], Cho et al. [65], Kim et al. [68], Sharma et al. [76], Juncal et al. [84], Plemel et al. [100], Bae et al. [105]	>12 DD
De Jong et al. [31], Ali Said et al. [79], Caporossi et al. [88], Fine et al. [94], Han et al. [96], Wilkins et al. [102]	Other (>2 quadrants; N/A)

## Data Availability

Data are available on reasonable request to the corresponding authors.

## Appendix A

Documentation on the literature search for:

Subretinal haemorrhage

Search date: 14 June 2022

The following databases were searched:

**Database**	**Number of Retrieved References**
Ovid Medline	1312
Embase	1411
Cochrane Central Register of Controlled Trials	22
Cochrane Database of Systematic Reviews	0
Number of references before deduplication:	2745
Number of references after deduplication:	1654

Search syntax:

/	After an index term indicates a subject heading was selected
.ti,ab,kf.	Search for a term in title, abstract, and author keywords
*	At the end of a term indicates that this term has been truncated; hemorrhag* retrieves haemorrhage, haemorrhages
Adj2	ADJ = adjacent operator in the Ovid databases. Adj2: search for two terms next to each other, in any order, up to 2 words in between.
NEAR/3	NEAR = proximity operator in the Cochrane Database of Systematic Reviews. NEAR/3: search for two terms next to each other, in any order, up to 2 words in between.

Search strategies:

Ovid MEDLINE(R) ALL 1946 to 8 June 2022

**#**	**Searches**	**Results**
1	((subretin* or sub-retin* or submacula* or sub-macula*) adj2 (hemorrhag* or haemorrhag* or bleed*)).ti,ab,kf.	1346
2	Retinal Haemorrhage/	5523
3	(subretin* or sub-retin* or submacula* or sub-macula*).ti,ab,kf.	11,699
4	1 or (2 and 3)	1509
5	limit 4 to english language	1312

Embase Classic + Embase 1947 to 8 June 2022

**#**	**Searches**	**Results**
1	((subretin* or sub-retin* or submacula* or sub-macula*) adj2 (hemorrhag* or haemorrhag* or bleed*)).ti,ab,kf.	1672
2	retina haemorrhage/ or retina macula haemorrhage/	10,977
3	(subretin* or sub-retin* or submacula* or sub-macula*).ti,ab,kf.	16,004
4	1 or (2 and 3)	2098
5	limit 4 to conference abstracts	234
6	4 not 5	1864
7	limit 6 to (article or review)	1725
8	limit 7 to english language	1411

Cochrane Central Register of Controlled Trials

1	((subretin* or sub-retin* or submacula* or sub-macula*) AND (hemorrhag* or haemorrhag* or bleed*)): in Record Title	22

Cochrane Database of Systematic Reviews

1	((subretin* or sub-retin* or submacula* or sub-macula*) NEAR/3 (hemorrhag* or haemorrhag* or bleed*)): in ti,ab,kw.	0

## Appendix B

Summary of the 155 records screened for inclusion/exclusion and the determining reasons behind each choice.

	**Title**	**Included (N. of Eyes)**	**Excluded**	**Explanation for Exclusion (or Comments)**
1	Olivier S, Chow DR, Packo KH, MacCumber MW, Awh CC. Subretinal recombinant tissue plasminogen activator injection and pneumatic displacement of thick submacular haemorrhage in Age-Related macular degeneration. Ophthalmology. 2004 Jun;111(6):1201–8. doi: 10.1016/j.ophtha.2003.10.020. Erratum in: Ophthalmology. 2004 Sep;111(9):1640. PMID: 15177972.	1 included (29 eyes)		
2	Woo, J. John M.D.; Lou, Peter L. M.D.; Ryan, Edward A. M.D.; Kroll, Arnold J. M.D. Surgical Treatment of Submacular Haemorrhage in Age-Related Macular Degeneration. International Ophthalmology Clinics: Winter 2004-Volume 44-Issue 1-p 43–50		Excluded	Review
3	Chan, W.-M., Liu, D.T., Lai, T.Y., Li, H., Tong, J.-P. and Lam, D.S. (2005), Extensive submacular haemorrhage in polypoidal choroidal vasculopathy managed by sequential gas displacement and photodynamic therapy: a pilot study of one-year follow up. Clinical & Experimental Ophthalmology, 33: 611–618. https://doi-org.ezproxy.uio.no/10.1111/j.1442-9071.2005.01105.x		Excluded	Pilot study, 6 eyes
4	Puchta, Koch, F., & Hattenbach, L. (2005). Prospektive, randomisierte Studie zur intravitrealen Gabe von rt-PA mit SF6-Gas vs. SF6-Gas in der Behandlung von submakulären Blutungen bei AMD. Klinische Monatsblätter für Augenheilkunde. https://doi.org/10.1055/s-2005-922145		Excluded	Languages other than English
5	Ratanasukon, M., Kittantong, A. Results of intravitreal tissue plasminogen activator and expansile gas injection for submacular haemorrhage in Thais. Eye 19, 1328–1332 (2005). https://doi.org/10.1038/sj.eye.6701769	2 included (24 eyes)		
6	Thompson JT, Sjaarda RN. Vitrectomy for the treatment of submacular haemorrhages from macular degeneration: a comparison of submacular haemorrhage/membrane removal and submacular tissue plasminogen activator-assisted pneumatic displacement. Trans Am Ophthalmol Soc. 2005;103:98–107; discussion 107. PMID: 17057793; PMCID: PMC1447564.	3 included (42 eyes)		
7	Wu TT, Sheu SJ. Intravitreal tissue plasminogen activator and pneumatic displacement of submacular haemorrhage secondary to retinal artery macroaneurysm. J Ocul Pharmacol Ther. 2005 Feb;21(1):62–7. doi: 10.1089/jop.2005.21.62. PMID: 15718829.		Excluded	Case series of only 6 eyes
8	Yang PM, Kuo HK, Kao ML, Chen YJ, Tsai HH. Pneumatic displacement of a dense submacular haemorrhage with or without tissue plasminogen activator. Chang Gung Med J. 2005 Dec;28(12):852–9. PMID: 16515019.	4 included (24 eyes)		
9	Mozaffarieh M, Heinzl H, Sacu S, Wedrich A. In-patient management and treatment satisfaction after intravitreous plasminogen activator injection. Graefes Arch Clin Exp Ophthalmol. 2006 Nov;244(11):1421–8. doi: 10.1007/s00417-005-0232-z. Epub 2006 Apr 5. PMID: 16596407.	5 included (101 eyes)		(No anatomical parameters)
10	Oie Y, Emi K. Surgical excision of retinal macroaneurysms with submacular haemorrhage. Jpn J Ophthalmol. 2006 Nov-Dec;50(6):550–553. doi: 10.1007/s10384-006-0369-2. Epub 2006 Dec 18. PMID: 17180532.		Excluded	Only 2 patients
11	Singh RP, Patel C, Sears JE. Management of subretinal macular haemorrhage by direct administration of tissue plasminogen activator. Br J Ophthalmol. 2006 Apr;90(4):429–31. doi: 10.1136/bjo.2005.085001. PMID: 16547320; PMCID: PMC1856980.	6 included (17 eyes)		
12	Chen CY, Hooper C, Chiu D, Chamberlain M, Karia N, Heriot WJ. Management of submacular haemorrhage with intravitreal injection of tissue plasminogen activator and expansile gas. Retina. 2007 Mar;27(3):321–8. doi: 10.1097/01.iae.0000237586.48231.75. PMID: 17460587.	7 included (104 eyes)		
13	Gopalakrishan M, Giridhar A, Bhat S, Saikumar SJ, Elias A, N S. Pneumatic displacement of submacular haemorrhage: safety, efficacy, and patient selection. Retina. 2007 Mar;27(3):329–34. doi: 10.1097/01.iae.0000231544.43093.40. PMID: 17460588.	8 included (20 eyes)		
14	Hasler PW, la Cour M, Villumsen J. Pneumatic displacement and intravitreal bevacizumab in the management of subretinal haemorrhage caused by choroidal neovascularization. Acta Ophthalmol Scand. 2007 Aug;85(5):577–9. doi: 10.1111/j.1600-0420.2007.00914.x. Epub 2007 Jun 8. PMID: 17559558.		Excluded	Case report
15	Oshima Y, Ohji M, Tano Y. Pars plana vitrectomy with peripheral retinotomy after injection of preoperative intravitreal tissue plasminogen activator: a modified procedure to drain massive subretinal haemorrhage. Br J Ophthalmol. 2007 Feb;91(2):193–8. doi: 10.1136/bjo.2006.101444. Epub 2006 Aug 17. PMID: 16916872; PMCID: PMC1857597.		Excluded	Surgical technique
16	Ron Y, Ehrlich R, Axer-Siegel R, Rosenblatt I, Weinberger D. Pneumatic displacement of submacular haemorrhage due to age-related macular degeneration. Ophthalmologica. 2007;221(1):57–61. doi: 10.1159/000096524. PMID: 17183203.	9 included (24 eyes)		
17	Stifter E, Michels S, Prager F, Georgopoulos M, Polak K, Hirn C, Schmidt-Erfurth U. Intravitreal bevacizumab therapy for neovascular age-related macular degeneration with large submacular haemorrhage. Am J Ophthalmol. 2007 Dec;144(6):886–892. doi: 10.1016/j.ajo.2007.07.034. Epub 2007 Oct 4. PMID: 17916314.	10 included (21 eyes)		
18	Liu, W. Current management of submacular haemorrhage in age-related macular degeneration. International Journal of Ophthalmology-Volume 8, Issue 0, pp. 867–870-published 2008-01-01		Excluded	Review
19	Meyer CH, Scholl HP, Eter N, Helb HM, Holz FG. Combined treatment of acute subretinal haemorrhages with intravitreal recombined tissue plasminogen activator, expansile gas and bevacizumab: a retrospective pilot study. Acta Ophthalmol. 2008 Aug;86(5):490–4. doi: 10.1111/j.1600-0420.2007.01125.x. Epub 2008 Jan 24. PMID: 18221499.	11 included (19 eyes)		
20	Nakamura H, Hayakawa K, Sawaguchi S, Gaja T, Nagamine N, Medoruma K. Visual outcome after vitreous, sub-internal limiting membrane, and/or submacular haemorrhage removal associated with ruptured retinal arterial macroaneurysms. Graefes Arch Clin Exp Ophthalmol. 2008 May;246(5):661–9. doi: 10.1007/s00417-007-0724-0. Epub 2007 Dec 11. PMID: 18071732.		Excluded	SRMH secondary to RAM
21	Chawla S, Misra V, Khemchandani M. Pneumatic displacement and intravitreal bevacizumab: a new approach for management of submacular haemorrhage in choroidal neovascular membrane. Indian J Ophthalmol. 2009 Mar-Apr;57(2):155–7. doi: 10.4103/0301-4738.45511. PMID: 19237795; PMCID: PMC2684421.		Excluded	Only 4 cases
22	Fang IM, Lin YC, Yang CH, Yang CM, Chen MS. Effects of intravitreal gas with or without tissue plasminogen activator on submacular haemorrhage in age-related macular degeneration. Eye (Lond). 2009 Feb;23(2):397–406. doi: 10.1038/sj.eye.6703017. Epub 2007 Nov 2. PMID: 17975562.	12 included (53 eyes)		
23	Gibran SK, Romano MR, Wong D. Surgical management of massive submacular haemorrhage associated with age-related macular degeneration. Retin Cases Brief Rep. 2009 Fall;3(4):391–4. doi: 10.1097/ICB.0b013e31818a470e. PMID: 25389857.		Excluded	6 eyes
24	Kamei M, Tano Y. Tissue plasminogen activator-assisted vitrectomy: surgical drainage of submacular haemorrhage. Dev Ophthalmol. 2009;44:82–88. doi: 10.1159/000223948. Epub 2009 Jun 3. PMID: 19494655.		Excluded	12 eyes
25	Arias L, Monés J. Transconjunctival sutureless vitrectomy with tissue plasminogen activator, gas and intravitreal bevacizumab in the management of predominantly hemorrhagic age-related macular degeneration. Clin Ophthalmol. 2010 Feb 18;4:67–72. doi: 10.2147/opth.s8635. PMID: 20186279; PMCID: PMC2827187.	13 included (15 eyes)		
26	Cakir M, Cekiç O, Yilmaz OF. Pneumatic displacement of acute submacular haemorrhage with and without the use of tissue plasminogen activator. Eur J Ophthalmol. 2010 May-Jun;20(3):565–71. doi: 10.1177/112067211002000305. PMID: 20037915.	14 included (21 eyes)		
27	Fine HF, Iranmanesh R, Del Priore LV, Barile GR, Chang LK, Chang S, Schiff WM. Surgical outcomes after massive subretinal haemorrhage secondary to age-related macular degeneration. Retina. 2010 Nov-Dec;30(10):1588–94. doi: 10.1097/IAE.0b013e3181e2263c. PMID: 20856172.	15 included (15 eyes)		
28	Hillenkamp J, Surguch V, Framme C, Gabel VP, Sachs HG. Management of submacular haemorrhage with intravitreal versus subretinal injection of recombinant tissue plasminogen activator. Graefes Arch Clin Exp Ophthalmol. 2010 Jan;248(1):5–11. doi: 10.1007/s00417-009-1158-7. Epub 2009 Aug 11. PMID: 19669780.	16 included (18 + 29 eyes)		
29	Höhn F, Mirshahi A, Hattenbach LO. Kombinierte intravitreale Injektion von Bevacizumab und SF(6)-Gas bei AMD-assoziierter, submakulärer Hämorrhagie [Combined intravitreal injection of bevacizumab and SF6 gas for treatment of submacular haemorrhage secondary to age-related macular degeneration]. Ophthalmologe. 2010 Apr;107(4):328–32. German. doi: 10.1007/s00347-009-2004-3. PMID: 19669150.		Excluded	Language other than English
30	Kung YH, Wu TT, Hong MC, Sheu SJ. Intravitreal tissue plasminogen activator and pneumatic displacement of submacular haemorrhage. J Ocul Pharmacol Ther. 2010 Oct;26(5):469–74. doi: 10.1089/jop.2010.0066. PMID: 20925578.	17 included (46 eyes)		
31	McAllister IL, Chen SD, Patel JI, Fleming BL, Yu DY. Management of submacular haemorrhage in age-related macular degeneration with intravitreal tenecteplase. Br J Ophthalmol. 2010 Feb;94(2):260–1. doi: 10.1136/bjo.2009.158170. PMID: 20139293.		Excluded	8 eyes
32	McKibbin M, Papastefanou V, Matthews B, Cook H, Downey L. Ranibizumab monotherapy for sub-foveal haemorrhage secondary to choroidal neovascularisation in age-related macular degeneration. Eye (Lond). 2010 Jun;24(6):994–8. doi: 10.1038/eye.2009.271. Epub 2009 Nov 13. PMID: 19911016.		Excluded	12 eyes
33	Sandhu SS, Manvikar S, Steel DH. Displacement of submacular haemorrhage associated with age-related macular degeneration using vitrectomy and submacular rt-PA injection followed by intravitreal ranibizumab. Clin Ophthalmol. 2010 Jul 21;4:637–42. doi: 10.2147/opth.s10060. PMID: 20668667; PMCID: PMC2909894.	18 included (16 eyes)		
34	Treumer F, Klatt C, Roider J, Hillenkamp J. Subretinal coapplication of recombinant tissue plasminogen activator and bevacizumab for neovascular age-related macular degeneration with submacular haemorrhage. Br J Ophthalmol. 2010 Jan;94(1):48–53. doi: 10.1136/bjo.2009.164707. Epub 2009 Nov 27. PMID: 19946027.		Excluded	12 eyes
35	Georgalas I, Papaconstantinou D, Karagiannis D, Ladas I. Pneumatic displacement of acute submacular haemorrhage with and without the use of rt-PA. Eur J Ophthalmol. 2011 Mar-Apr;21(2):220; author reply 221. doi: 10.5301/ejo.2010.5685. PMID: 20853260.		Excluded	Comment to the editor
36	Guthoff R, Guthoff T, Meigen T, Goebel W. Intravitreous injection of bevacizumab, tissue plasminogen activator, and gas in the treatment of submacular haemorrhage in age-related macular degeneration. Retina. 2011 Jan;31(1):36–40. doi: 10.1097/IAE.0b013e3181e37884. PMID: 20921929.	19 included (38 eyes)		
37	Mizutani T, Yasukawa T, Ito Y, Takase A, Hirano Y, Yoshida M, Ogura Y. Pneumatic displacement of submacular haemorrhage with or without tissue plasminogen activator. Graefes Arch Clin Exp Ophthalmol. 2011 Aug;249(8):1153–7. doi: 10.1007/s00417-011-1649-1. Epub 2011 Mar 29. PMID: 21445629.	20 included (53 eyes)		
38	Moriyama M, Ohno-Matsui K, Shimada N, Hayashi K, Kojima A, Yoshida T, Tokoro T, Mochizuki M. Correlation between visual prognosis and fundus autofluorescence and optical coherence tomographic findings in highly myopic eyes with submacular haemorrhage and without choroidal neovascularization. Retina. 2011 Jan;31(1):74–80. doi: 10.1097/IAE.0b013e3181e91148. PMID: 21187733.		Excluded	Macular hemorrhage secondary to pathologic myopia
39	Shultz RW, Bakri SJ. Treatment for submacular haemorrhage associated with neovascular age-related macular degeneration. Semin Ophthalmol. 2011 Nov;26(6):361–71. doi: 10.3109/08820538.2011.585368. PMID: 22044334.		Excluded	Review
40	Steel DH, Sandhu SS. Submacular haemorrhages associated with neovascular age-related macular degeneration. Br J Ophthalmol. 2011 Aug;95(8):1051–7. doi: 10.1136/bjo.2010.182253. Epub 2010 Sep 2. PMID: 20813746.		Excluded	Review
41	Tognetto D, Skiadaresi E, Cecchini P, Ravalico G. Subretinal recombinant tissue plasminogen activator and pneumatic displacement for the management of subretinal haemorrhage occurring after anti-VEGF injections for wet AMD. Clin Ophthalmol. 2011;5:459–63. doi: 10.2147/OPTH.S15864. Epub 2011 Apr 13. PMID: 21573092; PMCID: PMC3090299.		Excluded	3 cases
42	Treumer F, Roider J, Hillenkamp J. Long-term outcome of subretinal coapplication of rt-PA and bevacizumab followed by repeated intravitreal anti-VEGF injections for neovascular AMD with submacular haemorrhage. Br J Ophthalmol. 2012 May;96(5):708–13. doi: 10.1136/bjophthalmol-2011-300655. Epub 2011 Dec 15. PMID: 22174095.	21 included (41 eyes)		
43	Wu TT, Kung YH, Hong MC. Vitreous haemorrhage complicating intravitreal tissue plasminogen activator and pneumatic displacement of submacular haemorrhage. Retina. 2011 Nov;31(10):2071–7. doi: 10.1097/IAE.0b013e31822528c8. PMID: 21817964.		Excluded	Aim out of the scope: to evaluate the clinical factors associated with vitreous hemorrhage
44	Hesgaard HB, Torkashvand M, la Cour M. Failure to detect an effect of pneumatic displacement in the management of submacular haemorrhage secondary to age-related macular degeneration: a retrospective case series. Acta Ophthalmol. 2012 Sep;90(6):e498–500. doi: 10.1111/j.1755-3768.2011.02352.x. Epub 2012 Jan 23. PMID: 22268661.		Excluded	Letter to the editor
45	Hesse, L. Intravitreale Injektionen. Ophthalmologe 109, 644–647 (2012). https://doi.org/10.1007/s00347-012-2565-4		Excluded	Language other than English
46	Hillenkamp J, Klettner A, Puls S, Treumer F, Roider J. Subretinale Koapplikation von rt-PA und Bevacizumab bei exsudativer altersbedingter Makuladegeneration mit submakulärer Blutung. Kompatibilität der Wirkstoffe und klinische Langzeitergebnisse [Subretinal co-application of rt-PA and bevacizumab for exudative AMD with submacular haemorrhage. Compatibility and clinical long-term results]. Ophthalmologe. 2012 Jul;109(7):648–56. German. doi: 10.1007/s00347-012-2564-5. PMID: 22752624.		Excluded	Language other than English
47	Cochrane Central Register of Controlled Trials Intravitreal versus submacular injection of rt-PA for acute submacular haemorrhages NTR3359 https://trialsearch.who.int/Trial2.aspx?TrialID=NTR3359, 2012 | added to CENTRAL: 31 March 2019 | 2019 Issue 3 accessed on 1 July 2023.		Excluded	Trial protocol
48	Sonmez K, Ozturk F, Ozcan PY. Treatment of multilevel macular haemorrhage secondary to retinal arterial macroaneurysm with submacular tissue plasminogen activator. Eur J Ophthalmol. 2012 Nov-Dec;22(6):1026–31. doi: 10.5301/ejo.5000140. Epub 2012 Mar 20. PMID: 22467586.		Excluded	SRMH secondary to RAM
49	Szurman P. Subretinale Chirurgie bei Massenblutung [Subretinal surgery for massive haemorrhage]. Ophthalmologe. 2012 Jul;109(7):657–64. German. doi: 10.1007/s00347-012-2566-3. PMID: 22814924.		Excluded	Language other than English
50	Tsymanava A, Uhlig CE. Intravitreal recombinant tissue plasminogen activator without and with additional gas injection in patients with submacular haemorrhage associated with age-related macular degeneration. Acta Ophthalmol. 2012 Nov;90(7):633–8. doi: 10.1111/j.1755-3768.2011.02115.x. Epub 2011 Feb 18. PMID: 21332673.	22 included (110 eyes)		
51	Ueda-Arakawa N, Tsujikawa A, Yamashiro K, Ooto S, Tamura H, Yoshimura N. Visual prognosis of eyes with submacular haemorrhage associated with exudative age-related macular degeneration. Jpn J Ophthalmol. 2012 Nov;56(6):589–98. doi: 10.1007/s10384-012-0191-y. Epub 2012 Oct 4. PMID: 23053632.	23 included (31 eyes)		
52	Injection of Lucentis (Ranibizumab) in the vitreous body of the eye after eye surgery and application of recombinant tissue plasminogen activator (rt-PA) in patients with submacular bleeding complications suffering from wet age-related macular degeneration (AMD) EUCTR2010-018637-21-DE https://trialsearch.who.int/Trial2.aspx?TrialID=EUCTR2010-018637-21-DE, 2013 | added to CENTRAL: 31 March 2019 | 2019 Issue 3		Excluded	Protocol clinical trial
53	Intravitreal rt-PA and C_3_F_8_ for the Treatment of Submacular Haemorrhage as a Complication of Neovascular Age-related Macular Degeneration NCT01835067 https://clinicaltrials.gov/show/NCT01835067, 2013 | added to CENTRAL: 31 May 2018 | 2018 Issue 5		Excluded	Protocol clinical trial
54	Cheung CM, Bhargava M, Xiang L, Mathur R, Mun CC, Wong D, Wong TY. Six-month visual prognosis in eyes with submacular haemorrhage secondary to age-related macular degeneration or polypoidal choroidal vasculopathy. Graefes Arch Clin Exp Ophthalmol. 2013 Jan;251(1):19–25. doi: 10.1007/s00417-012-2029-1. Epub 2012 May 26. PMID: 22638617.		Excluded	
55	Cho HJ, Koh KM, Kim HS, Lee TG, Kim CG, Kim JW. Anti-vascular endothelial growth factor monotherapy in the treatment of submacular haemorrhage secondary to polypoidal choroidal vasculopathy. Am J Ophthalmol. 2013 Sep;156(3):524–531.e1. doi: 10.1016/j.ajo.2013.04.029. Epub 2013 Jun 13. PMID: 23769197.	24 included (27 eyes)		
56	Cho HJ, Koh KM, Kim HS, Lee TG, Kim CG, Kim JW. Anti-vascular endothelial growth factor monotherapy in the treatment of submacular haemorrhage secondary to polypoidal choroidal vasculopathy. Am J Ophthalmol. 2013 Sep;156(3):524–531.e1. doi: 10.1016/j.ajo.2013.04.029. Epub 2013 Jun 13. PMID: 23769197.	25 included (17 eyes)		
57	Han L, Ma Z, Wang C, Dou H, Hu Y, Feng X, Xu Y, Yin Z, Wang X. Autologous transplantation of simple retinal pigment epithelium sheet for massive submacular haemorrhage associated with pigment epithelium detachment. Invest Ophthalmol Vis Sci. 2013 Jul 24;54(7):4956–63. doi: 10.1167/iovs.13-11957. PMID: 23744996.	26 included (14 eyes)		
58	Jain S, Kishore K, Sharma YR. Intravitreal anti-VEGF monotherapy for thick submacular haemorrhage of less than 1 week duration secondary to neovascular age-related macular degeneration. Indian J Ophthalmol. 2013 Sep;61(9):490–6. doi: 10.4103/0301-4738.119432. PMID: 24104707; PMCID: PMC3831764.	27 included (14 eyes)		
59	Kapran Z, Ozkaya A, Uyar OM. Hemorrhagic age-related macular degeneration managed with vitrectomy, subretinal injection of tissue plasminogen activator, gas tamponade, and upright positioning. Ophthalmic Surg Lasers Imaging Retina. 2013 Sep-Oct;44(5):471–6. doi: 10.3928/23258160-20130909-09. PMID: 24044710.		Excluded	10 eyes
60	Lumi X, Sulak M. Treatment of submacular haemorrhage in patients with neovascular age related macular degeneration. Coll Antropol. 2013 Apr;37 Suppl 1:223–6. PMID: 23837248.		Excluded	9 patients
61	Martel JN, Mahmoud TH. Subretinal pneumatic displacement of subretinal haemorrhage. JAMA Ophthalmol. 2013 Dec;131(12):1632–5. doi: 10.1001/jamaophthalmol.2013.5464. PMID: 24337559.		Excluded	Surgical technique
62	Mayer WJ, Hakim I, Haritoglou C, Gandorfer A, Ulbig M, Kampik A, Wolf A. Efficacy and safety of recombinant tissue plasminogen activator and gas versus bevacizumab and gas for subretinal haemorrhage. Acta Ophthalmol. 2013 May;91(3):274–8. doi: 10.1111/j.1755-3768.2011.02264.x. Epub 2011 Sep 22. PMID: 21952010.	28 included (45 eyes)		
63	Papavasileiou E, Steel DH, Liazos E, McHugh D, Jackson TL. Intravitreal tissue plasminogen activator, perfluoropropane (C_3_F_8_), and ranibizumab or photodynamic therapy for submacular haemorrhage secondary to wet age-related macular degeneration. Retina. 2013 Apr;33(4):846–53. doi: 10.1097/IAE.0b013e318271f278. PMID: 23400079.		Excluded	7 eyes
64	Rishi E, Gopal L, Rishi P, Sengupta S, Sharma T. Submacular haemorrhage: a study amongst Indian eyes. Indian J Ophthalmol. 2012 Nov-Dec;60(6):521–5. doi: 10.4103/0301-4738.103779. PMID: 23202390; PMCID: PMC3545128.	29 included (46 eyes)		
65	Mark Sherman, Charles Barr, Shlomit Schaal; Functional and Anatomical Outcomes of Tissue Plasminogen Activator (rt-PA) Treatment for Submacular Haemorrhage Associated with Exudative Macular Degeneration (ExAMD): A Comparative Analysis Between Intra-vitreal and Sub-retinal rt-PA injected Patients. Invest. Ophthalmol. Vis. Sci. 2013;54(15):3304.		Excluded	Meeting abstract
66	Shienbaum G, Garcia Filho CA, Flynn HW Jr, Nunes RP, Smiddy WE, Rosenfeld PJ. Management of submacular haemorrhage secondary to neovascular age-related macular degeneration with anti-vascular endothelial growth factor monotherapy. Am J Ophthalmol. 2013 Jun;155(6):1009–13. doi: 10.1016/j.ajo.2013.01.012. Epub 2013 Mar 7. PMID: 23465269.	30 included (19 eyes)		
67	van Zeeburg EJ, Cereda MG, van Meurs JC. Recombinant tissue plasminogen activator, vitrectomy, and gas for recent submacular haemorrhage displacement due to retinal macroaneurysm. Graefes Arch Clin Exp Ophthalmol. 2013 Mar;251(3):733–40. doi: 10.1007/s00417-012-2116-3. Epub 2012 Aug 4. PMID: 22865261.		Excluded	SRMH secondary to RAM
68	van Zeeburg EJ, van Meurs JC. Literature review of recombinant tissue plasminogen activator used for recent-onset submacular haemorrhage displacement in age-related macular degeneration. Ophthalmologica. 2013;229(1):1–14. doi: 10.1159/000343066. Epub 2012 Oct 12. PMID: 23075629.		Excluded	Review
69	Chang W, Garg SJ, Maturi R, Hsu J, Sivalingam A, Gupta SA, Regillo CD, Ho AC. Management of thick submacular haemorrhage with subretinal tissue plasminogen activator and pneumatic displacement for age-related macular degeneration. Am J Ophthalmol. 2014 Jun;157(6):1250–7. doi: 10.1016/j.ajo.2014.02.007. Epub 2014 Feb 13. PMID: 24531021.	31 included (101 eyes)		
70	Dewilde E, Delaere L, Vaninbroukx I, Van Calster J, Stalmans P. Subretinal tissue plasminogen activator injection to treat submacular haemorrhage during age-related macular degeneration. Acta Ophthalmol. 2014 Sep;92(6):e497–8. doi: 10.1111/aos.12458. Epub 2014 Jun 18. PMID: 24943231.		Excluded	Letter to the editor
71	Iacono P, Parodi MB, Introini U, La Spina C, Varano M, Bandello F. Intravitreal ranibizumab for choroidal neovascularization with large submacular haemorrhage in age-related macular degeneration. Retina. 2014 Feb;34(2):281–7. doi: 10.1097/IAE.0b013e3182979e33. PMID: 23851632.	32 included (23 eyes)		
72	Kitahashi M, Baba T, Sakurai M, Yokouchi H, Kubota-Taniai M, Mitamura Y, Yamamoto S. Pneumatic displacement with intravitreal bevacizumab for massive submacular haemorrhage due to polypoidal choroidal vasculopathy. Clin Ophthalmol. 2014 Mar 3;8:485–92. doi: 10.2147/OPTH.S55413. PMID: 24623972; PMCID: PMC3949732.	33 included (32 eyes)		
73	Gerard F McGowan, David Steel, David Yorston; AMD with submacular haemorrhage: new insights from a population-based study. Invest. Ophthalmol. Vis. Sci. 2014;55(13):662.		Excluded	Meeting abstract
74	Moisseiev E, Ben Ami T, Barak A. Vitrectomy and subretinal injection of tissue plasminogen activator for large submacular haemorrhage secondary to AMD. Eur J Ophthalmol. 2014 Nov-Dec;24(6):925–31. doi: 10.5301/ejo.5000500. Epub 2014 Jun 12. PMID: 24966031.	34 included (31 eyes)		
75	Dimopoulos S, Leitritz MA, Ziemssen F, Voykov B, Bartz-Schmidt KU, Gelisken F. Submacular predominantly hemorrhagic choroidal neovascularization: resolution of bleedings under anti-VEGF therapy. Clin Ophthalmol. 2015 Aug 24;9:1537–41. doi: 10.2147/OPTH.S87919. PMID: 26346691; PMCID: PMC4554429.	35 included (46 eyes)		
76	Hirashima T, Moriya T, Bun T, Utsumi T, Hirose M, Oh H. Optical coherence tomography findings and surgical outcomes of tissue plasminogen activator-assisted vitrectomy for submacular haemorrhage secondary to age-related macular degeneration. Retina. 2015 Oct;35(10):1969–78. doi: 10.1097/IAE.0000000000000574. PMID: 26079475.		Excluded	9 eyes
77	Inoue M, Shiraga F, Shirakata Y, Morizane Y, Kimura S, Hirakata A. Subretinal injection of recombinant tissue plasminogen activator for submacular haemorrhage associated with ruptured retinal arterial macroaneurysm. Graefes Arch Clin Exp Ophthalmol. 2015 Oct;253(10):1663–9. doi: 10.1007/s00417-014-2861-6. Epub 2014 Nov 25. PMID: 25418034.		Excluded	RAM
78	Prospective intervention study for drainage of subretinal haemorrhage using tissue plasminogen activator Authors: Jprn, Umin; Journal: https://trialsearch.who.int/Trial2.aspx?TrialID=JPRN-UMIN000019668		Excluded	Clinical trial protocol
79	Kadonosono K, Arakawa A, Yamane S, Inoue M, Yamakawa T, Uchio E, Yanagi Y. Displacement of submacular haemorrhages in age-related macular degeneration with subretinal tissue plasminogen activator and air. Ophthalmology. 2015 Jan;122(1):123–8. doi: 10.1016/j.ophtha.2014.07.027. Epub 2014 Sep 4. PMID: 25200400.	36 included (13 eyes)		
80	Kim HS, Cho HJ, Yoo SG, Kim JH, Han JI, Lee TG, Kim JW. Intravitreal anti-vascular endothelial growth factor monotherapy for large submacular haemorrhage secondary to neovascular age-related macular degeneration. Eye (Lond). 2015 Sep;29(9):1141–51. doi: 10.1038/eye.2015.131. Epub 2015 Aug 14. PMID: 26272443; PMCID: PMC4565949.	37 included (49 eyes)		
81	Kimura S, Morizane Y, Hosokawa M, Shiode Y, Kawata T, Doi S, Matoba R, Hosogi M, Fujiwara A, Inoue Y, Shiraga F. Submacular haemorrhage in polypoidal choroidal vasculopathy treated by vitrectomy and subretinal tissue plasminogen activator. Am J Ophthalmol. 2015 Apr;159(4):683–9. doi: 10.1016/j.ajo.2014.12.020. Epub 2014 Dec 30. PMID: 25555798.	38 included (15 eyes)		
82	Nayak S, Padhi TR, Basu S, Das T. Pneumatic displacement and intra-vitreal bevacizumab in management of sub-retinal and sub-retinal pigment epithelial haemorrhage at macula in polypoidal choroidal vasculopathy (PCV): rationale and outcome. Semin Ophthalmol. 2015 Jan;30(1):53–5. doi: 10.3109/08820538.2013.807849. Epub 2013 Aug 15. PMID: 23947424.		Excluded	3 eyes
83	Schaal, S.; Apenbrinck, E.; Barr, C. C.; Management of thick submacular haemorrhage with subretinal tissue plasminogen activator and pneumatic displacement for age-related macular degeneration. February 2015American Journal of Ophthalmology 159(2) DOI: 10.1016/j.ajo.2014.10.024		Excluded	Correspondence article
84	Shin JY, Lee JM, Byeon SH. Anti-vascular endothelial growth factor with or without pneumatic displacement for submacular haemorrhage. Am J Ophthalmol. 2015 May;159(5):904–14.e1. doi: 10.1016/j.ajo.2015.01.024. Epub 2015 Jan 28. PMID: 25637179.	39 included (82 eyes)		
85	Wei Y, Zhang Z, Jiang X, Li F, Zhang T, Qiu S, Yang Y, Zhang S. A surgical approach to large subretinal haemorrhage using pars plana vitrectomy and 360° retinotomy. Retina. 2015 Aug;35(8):1631–9. doi: 10.1097/IAE.0000000000000501. PMID: 26214315.	40 included (21 eyes)		
86	Abdelkader E, Yip KP, Cornish KS. Pneumatic displacement of submacular haemorrhage. Saudi J Ophthalmol. 2016 Oct-Dec;30(4):221–226. doi: 10.1016/j.sjopt.2016.10.002. Epub 2016 Oct 13. PMID: 28003779; PMCID: PMC5161816.		Excluded	12 eyes, 9 with SRMH secondary to AMD
87	Araújo J, Sousa C, Faria PA, Carneiro Â, Rocha-Sousa A, Falcão-Reis F. Intravitreal injection of recombinant tissue plasminogen activator in submacular haemorrhage: case series. Eur J Ophthalmol. 2016 Apr 12;26(3):e49–51. doi: 10.5301/ejo.5000682. PMID: 26428222.		Excluded	6 eyes
88	Bae K, Cho GE, Yoon JM, Kang SW. Optical Coherence Tomographic Features and Prognosis of Pneumatic Displacement for Submacular Haemorrhage. PLoS One. 2016 Dec 19;11(12):e0168474. doi: 10.1371/journal.pone.0168474. PMID: 27992524; PMCID: PMC5167395.	41 included (37 eyes)		
89	de Jong JH, van Zeeburg EJ, Cereda MG, van Velthoven ME, Faridpooya K, Vermeer KA, van Meurs JC. Intravitreal versus subretinal administration of recombinant tissue plasminogen activator combined with gas for acute submacular haemorrhages due to age-related macular degeneration: An Exploratory Prospective Study. Retina. 2016 May;36(5):914–25. doi: 10.1097/IAE.0000000000000954. PMID: 26807631.	42 included (24 eyes)		
90	de Silva SR, Bindra MS. Early treatment of acute submacular haemorrhage secondary to wet AMD using intravitreal tissue plasminogen activator, C_3_F_8_, and an anti-VEGF agent. Eye (Lond). 2016 Jul;30(7):952–7. doi: 10.1038/eye.2016.67. Epub 2016 Apr 15. PMID: 27080482; PMCID: PMC4941069.		Excluded	8 eyes
91	Dhawan, B.; Vig, V.; Singh, P.; Singh, R.; Management of sub macular haemorrhage with intravitreal injection of tissue plasminogen activator and sulfur hexafluoride. Journal Retina-Vitreus		Excluded	Not retrievable
92	Fassbender JM, Sherman MP, Barr CC, Schaal S. Tissue plasminogen activator for subfoveal haemorrhage due to age-related macular degeneration: Comparison of 3 Treatment Modalities. Retina. 2016 Oct;36(10):1860–5. doi: 10.1097/IAE.0000000000001030. PMID: 26945238.	43 included (39 eyes)		
93	González-López JJ, McGowan G, Chapman E, Yorston D. Vitrectomy with subretinal tissue plasminogen activator and ranibizumab for submacular haemorrhages secondary to age-related macular degeneration: retrospective case series of 45 consecutive cases. Eye (Lond). 2016 Jul;30(7):929–35. doi: 10.1038/eye.2016.65. Epub 2016 Apr 8. PMID: 27055681; PMCID: PMC4941067.	44 included (45 eyes)		
94	Isizaki E, Morishita S, Sato T, Fukumoto M, Suzuki H, Kida T, Ueki M, Ikeda T. Treatment of massive subretinal hematoma associated with age-related macular degeneration using vitrectomy with intentional giant tear. Int Ophthalmol. 2016 Apr;36(2):199–206. doi: 10.1007/s10792-015-0102-6. Epub 2015 Jul 28. PMID: 26216161.		Excluded	12 eyes
95	Kitagawa Y, Shimada H, Mori R, Tanaka K, Yuzawa M. Intravitreal Tissue Plasminogen Activator, Ranibizumab, and Gas Injection for Submacular Haemorrhage in Polypoidal Choroidal Vasculopathy. Ophthalmology. 2016 Jun;123(6):1278–86. doi: 10.1016/j.ophtha.2016.01.035. Epub 2016 Mar 2. PMID: 26949121.	45 included (20 eyes)		
96	Kumar A, Roy S, Bansal M, Tinwala S, Aron N, Temkar S, Pujari A. Modified Approach in Management of Submacular Haemorrhage Secondary to Wet Age-Related Macular Degeneration. Asia Pac J Ophthalmol (Phila). 2016 Mar-Apr;5(2):143–6. doi: 10.1097/APO.0000000000000130. PMID: 26302314.		Excluded	10 eyes
97	Lee JP, Park JS, Kwon OW, You YS, Kim SH. Management of Acute Submacular Haemorrhage with Intravitreal Injection of Tenecteplase, Anti-vascular Endothelial Growth Factor and Gas. Korean J Ophthalmol. 2016 Jun;30(3):192–7. doi: 10.3341/kjo.2016.30.3.192. Epub 2016 May 18. PMID: 27247518; PMCID: PMC4878979.	46 included (25 eyes)		
98	Lin TC, Hwang DK, Lee FL, Chen SJ. Visual prognosis of massive submacular haemorrhage in polypoidal choroidal vasculopathy with or without combination treatment. J Chin Med Assoc. 2016 Mar;79(3):159–65. doi: 10.1016/j.jcma.2015.11.004. Epub 2016 Jan 8. PMID: 26775600.	47 included (20 eyes)		
99	Liu H, Zhang LY, Li XX, Wu MQ. 23-Gauge vitrectomy with external drainage therapy as a novel procedure to displace massive submacular haemorrhage secondary to polypoidal choroidal vasculopathy. Medicine (Baltimore). 2016 Aug;95(32):e4192. doi: 10.1097/MD.0000000000004192. PMID: 27512837; PMCID: PMC4985292.		Excluded	4 eyes
100	Sadeghi Y, Elalouf M, Mantel I, Pournaras JA. Vitrectomy with Gas Tamponade and anti-VEGF Injections for the Management of Submacular Haemorrhage. Klin Monbl Augenheilkd. 2016 Apr;233(4):500–2. English. doi: 10.1055/s-0042-102567. Epub 2016 Apr 26. PMID: 27116519.		Excluded	Case report
101	Stanescu-Segall D, Balta F, Jackson TL. Submacular haemorrhage in neovascular age-related macular degeneration: A synthesis of the literature. Surv Ophthalmol. 2016 Jan-Feb;61(1):18–32. doi: 10.1016/j.survophthal.2015.04.004. Epub 2015 Jul 23. PMID: 26212151.		Excluded	Review
102	Waizel M, Todorova MG, Kazerounian S, Rickmann A, Blanke BR, Szurman P. Efficacy of Vitrectomy Combined with Subretinal Recombinant Tissue Plasminogen Activator for Subretinal versus Subpigment Epithelial versus Combined Haemorrhages. Ophthalmologica. 2016;236(3):123–132. doi: 10.1159/000449172. Epub 2016 Sep 16. PMID: 27631507.	48 included (19 eyes)		
103	Bell JE, Shulman JP, Swan RJ, Teske MP, Bernstein PS. Intravitreal Versus Subretinal Tissue Plasminogen Activator Injection for Submacular Haemorrhage. Ophthalmic Surg Lasers Imaging Retina. 2017 Jan 1;48(1):26–32. doi: 10.3928/23258160-20161219-04. PMID: 28060391.	49 included (18 eyes)		
104	Fleissig E, Barak A, Goldstein M, Loewenstein A, Schwartz S. Massive subretinal and subretinal pigment epithelial haemorrhage displacement with perfluorocarbon liquid using a two-step vitrectomy technique. Graefes Arch Clin Exp Ophthalmol. 2017 Jul;255(7):1341–1347. doi: 10.1007/s00417-017-3648-3. Epub 2017 Apr 15. PMID: 28412773.		Excluded	7 eyes
105	Fotis K, Garcia-Cabrera R, Ohn M, Chandra A. Anatomical and Functional Outcome of Pars Plana Vitrectomy and Subretinal Recombinant Tissue Plasminogen Activator for a Macular Subpigment Epithelial Haemorrhage. Ophthalmologica. 2017;238(1–2):106–108. doi: 10.1159/000475891. Epub 2017 May 24. PMID: 28535542.		Excluded	Case report
106	Gok M, Karabaş VL, Aslan MS, Kara Ö, Karaman S, Yenihayat F. Tissue plasminogen activator-assisted vitrectomy for submacular haemorrhage due to age-related macular degeneration. Indian J Ophthalmol. 2017 Jun;65(6):482–487. doi: 10.4103/ijo.IJO_129_16. PMID: 28643713; PMCID: PMC5508459.	50 included (17 eyes)		
107	Kimura S, Morizane Y, Matoba R, Hosokawa M, Shiode Y, Hirano M, Doi S, Toshima S, Takahashi K, Hosogi M, Fujiwara A, Shiraga F. Retinal sensitivity after displacement of submacular haemorrhage due to polypoidal choroidal vasculopathy: effectiveness and safety of subretinal tissue plasminogen activator. Jpn J Ophthalmol. 2017 Nov;61(6):472–478. doi: 10.1007/s10384-017-0530-0. Epub 2017 Aug 23. PMID: 28836011.		Excluded	11 eyes
108	Amy Q. Lu, Jay G. Prensky, Paul S. Baker, Ingrid U. Scott, Tamer H. Mahmoud, Bozho Todorich. (2020) Update on medical and surgical management of submacular haemorrhage. Expert Review of Ophthalmology 15:1, pages 43–57.		Excluded	Review
109	Tan, C.S., Lim, L.W. & Ngo, W.K. Treatment of massive subretinal haemorrhage from polypoidal choroidal vasculopathy and age-related macular degeneration. Int Ophthalmol 37, 779–780 (2017). https://doi.org/10.1007/s10792-016-0351-z		Excluded	Letter to the editor
110	Waizel M, Todorova MG, Rickmann A, Blanke BR, Szurman P. Efficacy of Vitrectomy Combined with Subretinal rt-PA Injection with Gas or Air Tamponade. Klin Monbl Augenheilkd. 2017 Apr;234(4):487–492. English. doi: 10.1055/s-0042-121575. Epub 2017 Jan 31. PMID: 28142164.	51 included (85 eyes)		
111	Waizel M, Todorova MG, Rickmann A, Blanke BR, Szurman P. Structural and Functional Outcome of Vitrectomy Combined with Subretinal Recombinant Tissue Plasminogen Activator for Isolated Subpigment Epithelial Haemorrhages. Ophthalmologica. 2017;238(1–2):109. doi: 10.1159/000475892. Epub 2017 May 24. PMID: 28535505.		Excluded	Letter to the editor
112	Cochrane Central Register of Controlled Trials Impacts of pneumatic displacement of submacular haemorrhage secondary to age-related macular degeneration on retinal pigment epithelial detachment UMIN000031065 https://trialsearch.who.int/Trial2.aspx?TrialID=JPRN-UMIN000031065, 2018 | added to CENTRAL: 31 March 2019 | 2019 Issue 3 Sourced from: ICTRP Links: WHO ICTRP		Excluded	Trial registration
113	Bardak H, Bardak Y, Erçalık Y, Erdem B, Arslan G, Timlioglu S. Sequential tissue plasminogen activator, pneumatic displacement, and anti-VEGF treatment for submacular haemorrhage. Eur J Ophthalmol. 2018 May;28(3):306–310. doi: 10.5301/ejo.5001074. PMID: 29148027.	52 included (16 eyes)		
114	Juncal VR, Hanout M, Altomare F, Chow DR, Giavedoni LR, Muni RH, Wong DT, Berger AR. Surgical management of submacular haemorrhage: experience at an academic Canadian centre. Can J Ophthalmol. 2018 Aug;53(4):408–414. doi: 10.1016/j.jcjo.2017.10.010. Epub 2017 Dec 23. PMID: 30119797.	53 included (99 eyes)		
115	Kim JH, Chang YS, Lee DW, Kim CG, Kim JW. Quantification of retinal changes after resolution of submacular haemorrhage secondary to polypoidal choroidal vasculopathy. Jpn J Ophthalmol. 2018 Jan;62(1):54–62. doi: 10.1007/s10384-017-0549-2. Epub 2017 Nov 29. PMID: 29188462.	54 included (21 eyes)		
116	Management of Submacular Haemorrhage in Age-Related Macular Degeneration Authors: Kim, L. A.; Eliott, D.; Journal: Ophthalmology Retina-Volume 2, Issue 3, pp. 177–179-published 2018-01-01		Excluded	Review
117	Kimura M, Yasukawa T, Shibata Y, Kato A, Hirano Y, Uemura A, Yoshida M, Ogura Y. Flattening of retinal pigment epithelial detachments after pneumatic displacement of submacular haemorrhages secondary to age-related macular degeneration. Graefes Arch Clin Exp Ophthalmol. 2018 Oct;256(10):1823–1829. doi: 10.1007/s00417-018-4059-9. Epub 2018 Jul 1. PMID: 29961921.	55 included (33 eyes)		
118	Ozkaya A, Erdogan G, Tarakcioglu HN. Submacular haemorrhage secondary to age-related macular degeneration managed with vitrectomy, subretinal injection of tissue plasminogen activator, haemorrhage displacement with liquid perfluorocarbon, gas tamponade, and face-down positioning. Saudi J Ophthalmol. 2018 Oct-Dec;32(4):269–274. doi: 10.1016/j.sjopt.2018.08.002. Epub 2018 Aug 15. PMID: 30581295; PMCID: PMC6300785.		Excluded	9 eyes
119	Sharma S, Kumar JB, Kim JE, Thordsen J, Dayani P, Ober M, Mahmoud TH. Pneumatic Displacement of Submacular Haemorrhage with Subretinal Air and Tissue Plasminogen Activator: Initial United States Experience. Ophthalmol Retina. 2018 Mar;2(3):180–186. doi: 10.1016/j.oret.2017.07.012. Epub 2017 Sep 28. PMID: 31047581.	56 included (24 eyes)		
120	Adrean SD, Chaili S, Pirouz A, Grant S. Rapid displacement of subretinal haemorrhage from a choroidal neovascular membrane with intravitreal C_3_F_8_ gas and face-down positioning. Am J Ophthalmol Case Rep. 2019 Mar 14;14:79–82. doi: 10.1016/j.ajoc.2019.03.003. PMID: 30949612; PMCID: PMC6428934.		Excluded	Case report
121	Balughatta P, Kadri V, Braganza S, Jayadev C, Mehta RA, Nakhate V, Yadav NK, Shetty R. Pneumatic displacement of limited traumatic submacular haemorrhage without tissue plasminogen activator: a case series. Retin Cases Brief Rep. 2019 Winter;13(1):34–38. doi: 10.1097/ICB.0000000000000525. PMID: 28079650.		Excluded	Case report
122	Gujral GS, Agarwal M, Mayor R, Shroff D, Chhablani J, Shanmugam MP. Clinical profile and management outcomes of traumatic submacular haemorrhage. J Curr Ophthalmol. 2019 Oct 22;31(4):411–415. doi: 10.1016/j.joco.2019.09.001. PMID: 31844792; PMCID: PMC6896465.		Excluded	Traumatic SRMH
123	Li ZX, Hu YJ, Atik A, Lu L, Hu J. Long-term observation of vitrectomy without subretinal haemorrhage management for massive vitreous haemorrhage secondary to polypoidal choroidal vasculopathy. Int J Ophthalmol. 2019 Dec 18;12(12):1859–1864. doi: 10.18240/ijo.2019.12.07. PMID: 31850169; PMCID: PMC6901888.		Excluded	Vitreous hemorrhage without SRMH
124	Plemel DJA, Lapere SRJ, Rudnisky CJ, Tennant MTS. VITRECTOMY WITH SUBRETINAL TISSUE PLASMINOGEN ACTIVATOR AND GAS TAMPONADE FOR SUBFOVEAL HAEMORRHAGE: Prognostic Factors and Clinical Outcomes. Retina. 2019 Jan;39(1):172–179. doi: 10.1097/IAE.0000000000001931. PMID: 29135798.	57 included (78 eyes)		
125	Erdogan G, Kirmaci A, Perente I, Artunay O. Gravitational displacement of submacular haemorrhage in patients with age-related macular disease. Eye (Lond). 2020 Jun;34(6):1136–1141. doi: 10.1038/s41433-019-0720-8. Epub 2019 Dec 2. PMID: 31792350; PMCID: PMC7253466.		Excluded	9 eyes
126	Helaiwa K, Paez LR, Szurman P, Januschowski K. Combined Administration of Preoperative Intravitreal and Intraoperative Subretinal Recombinant Tissue Plasminogen Activator in Acute Hemorrhagic Age-related Macular Degeneration. Cureus. 2020 Mar 10;12(3):e7229. doi: 10.7759/cureus.7229. PMID: 32190528; PMCID: PMC7065728.	58 included (14 eyes)		
127	Jeong S, Park DG, Sagong M. Management of a Submacular Haemorrhage Secondary to Age-Related Macular Degeneration: A Comparison of Three Treatment Modalities. J Clin Med. 2020 Sep 24;9(10):3088. doi: 10.3390/jcm9103088. PMID: 32987903; PMCID: PMC7601376.	59 included (77 eyes)		
128	Karamitsos A, Papastavrou V, Ivanova T, Cottrell D, Stannard K, Karachrysafi S, Cheristanidis S, Ziakas N, Papamitsou T, Hillier R. Management of acute submacular haemorrhage using intravitreal injection of tissue plasminogen activator and gas: A case series. SAGE Open Med Case Rep. 2020 Nov 13;8:2050313X20970337. doi: 10.1177/2050313X20970337. PMID: 33240500; PMCID: PMC7675899.	60 included (28 eyes)		
129	Kim JH, Kim CG, Lee DW, Yoo SJ, Lew YJ, Cho HJ, Kim JY, Lee SH, Kim JW. Intravitreal aflibercept for submacular haemorrhage secondary to neovascular age-related macular degeneration and polypoidal choroidal vasculopathy. Graefes Arch Clin Exp Ophthalmol. 2020 Jan;258(1):107–116. doi: 10.1007/s00417-019-04474-0. Epub 2019 Nov 18. PMID: 31741044.	61 included (29 eyes)		
130	Lee K, Park YG, Park YH. Visual prognosis after pneumatic displacement of submacular haemorrhage according to age-related macular degeneration subtypes. Retina. 2020 Dec;40(12):2304–2311. doi: 10.1097/IAE.0000000000002762. PMID: 31985556.	62 included (67 eyes)		
131	Amy Q. Lu, Jay G. Prensky, Paul S. Baker, Ingrid U. Scott, Tamer H. Mahmoud & Bozho Todorich (2020) Update on medical and surgical management of submacular haemorrhage, Expert Review of Ophthalmology, 15:1, 43–57, DOI: 10.1080/17469899.2020.1725474		Excluded	Review
132	Maggio E, Peroglio Deiro A, Mete M, Sartore M, Polito A, Prigione G, Guerriero M, Pertile G. Intravitreal Recombinant Tissue Plasminogen Activator and Sulphur Hexafluoride Gas for Submacular Haemorrhage Displacement in Age-Related Macular Degeneration: Looking behind the Blood. Ophthalmologica. 2020;243(3):224–235. doi: 10.1159/000505752. Epub 2020 Jan 7. PMID: 31905361.	63 included (96)		
133	Onder Tokuc, E.; Levent Karabas, V.; Surgical management of subretinal haemorrhage. Journal: Retina-Vitreus-Volume 29, Issue 0, pp. 1–9-published 2020-01-01		Excluded	Review
134	Sun, T.; Wan, Z.; Gao, Y.; Zhang, L.; Peng, Q. Fundus imaging features of massive hemorrhaging in polypoidal choroidal vasculopathy after treatment. International Journal of Clinical and Experimental Medicine-Volume 13, Issue 0, pp. 5736–5744-published 2020-01-01		Excluded	Case series of 9 eyes
135	Wilkins CS, Mehta N, Wu CY, Barash A, Deobhakta AA, Rosen RB. Outcomes of pars plana vitrectomy with subretinal tissue plasminogen activator injection and pneumatic displacement of fovea-involving submacular haemorrhage. BMJ Open Ophthalmol. 2020 Mar 16;5(1):e000394. doi: 10.1136/bmjophth-2019-000394. PMID: 32201733; PMCID: PMC7076260.	64 included (37 eyes)		
136	Ali Said Y, Dewilde E, Stalmans P. Visual Outcome after Vitrectomy with Subretinal rt-PA Injection to Treat Submacular Haemorrhage Secondary to Age-Related Macular Degeneration or Macroaneurysm. J Ophthalmol. 2021 Dec 30;2021:3160963. doi: 10.1155/2021/3160963. PMID: 35003789; PMCID: PMC8736698.	65 included (93 eyes)		
137	Avcı R, Mavi Yıldız A, Çınar E, Yılmaz S, Küçükerdönmez C, Akalp FD, Avcı E. Subretinal Coapplication of Tissue Plasminogen Activator and Bevacizumab with Concurrent Pneumatic Displacement for Submacular Haemorrhages Secondary to Neovascular Age-Related Macular Degeneration. Turk J Ophthalmol. 2021 Feb 25;51(1):38–44. doi: 10.4274/tjo.galenos.2020.72540. PMID: 33631914; PMCID: PMC7931654.	66 included (30 patients)		
138	Caporossi T, Bacherini D, Governatori L, Oliverio L, Di Leo L, Tartaro R, Rizzo S. Management of submacular massive haemorrhage in age-related macular degeneration: comparison between subretinal transplant of human amniotic membrane and subretinal injection of tissue plasminogen activator. Acta Ophthalmol. 2022 Aug;100(5):e1143–e1152. doi: 10.1111/aos.15045. Epub 2021 Oct 5. PMID: 34609787.	67 included (44 eyes)		
139	Iannetta D, De Maria M, Bolletta E, Mastrofilippo V, Moramarco A, Fontana L. Subretinal Injection of Recombinant Tissue Plasminogen Activator and Gas Tamponade to Displace Acute Submacular Haemorrhages Secondary to Age-Related Macular Degeneration. Clin Ophthalmol. 2021;15:3649–3659 https://doi.org/10.2147/OPTH.S324091	68 included (25 eyes)		
140	Iyer PG, Brooks HL Jr, Flynn HW Jr. Long-Term Favorable Visual Outcomes in Patients with Large Submacular Haemorrhage. Clin Ophthalmol. 2021;15:1189–1192 https://doi.org/10.2147/OPTH.S300662		Excluded	Case series (2 eyes)
141	Aslı Kırmacı Kabakcı, Gürkan Erdoğan, Burcu Kemer Atik, İrfan Perente. Outcomes of Surgical Treatment in Cases with Submacular Haemorrhage.	69 included (54 eyes)		
142	Kawakami S, Wakabayashi Y, Umazume K, Usui Y, Muramatsu D, Agawa T, Yamamoto K, Goto H. Long-Term Outcome of Eyes with Vitrectomy for Submacular and/or Vitreous Haemorrhage in Neovascular Age-Related Macular Degeneration. J Ophthalmol. 2021 Nov 2;2021:2963822. doi: 10.1155/2021/2963822. PMID: 34765261; PMCID: PMC8577947.	70 included (25 eyes)		
143	Kishikova L, Saad AAA, Vaideanu-Collins D, Isac M, Hamada D, El-Haig WM. Comparison between different techniques for treatment of submacular haemorrhage due to Age-Related Macular Degeneration. Eur J Ophthalmol. 2021 Sep;31(5):2621–2624. doi: 10.1177/1120672120959551. Epub 2020 Sep 29. PMID: 32993349.	71 included (29 eyes)		
144	Pierre M, Mainguy A, Chatziralli I, Pakzad-Vaezi K, Ruiz-Medrano J, Bodaghi B, Loewenstein A, Ambati J, de Smet MD, Tadayoni R, Touhami S. Macular Haemorrhage Due to Age-Related Macular Degeneration or Retinal Arterial Macroaneurysm: Predictive Factors of Surgical Outcome. J Clin Med. 2021 Dec 10;10(24):5787. doi: 10.3390/jcm10245787. PMID: 34945083; PMCID: PMC8703651.	72 included (65 eyes)		
145	Matsuo Y, Haruta M, Ishibashi Y, Ishibashi K, Furushima K, Kato N, Murotani K, Yoshida S. Visual Outcomes and Prognostic Factors of Large Submacular Haemorrhages Secondary to Polypoidal Choroidal Vasculopathy. Clin Ophthalmol. 2021 Aug 24;15:3557–3562. doi: 10.2147/OPTH.S327138. PMID: 34465976; PMCID: PMC8403222.	73 included (30 eyes)		
146	Rickmann A, Paez LR, Della Volpe Waizel M, Bisorca-Gassendorf L, Schulz A, Vandebroek AC, Szurman P, Januschowski K. Functional and structural outcome after vitrectomy combined with subretinal rt-PA Injection with or without additional intravitreal Bevacizumab injection for submacular haemorrhages. PLoS ONE. 2021 Apr 30;16(4):e0250587. doi: 10.1371/journal.pone.0250587. PMID: 33930041; PMCID: PMC8087026.	74 included (31 eyes)		
147	Saito-Uchida S, Inoue M, Koto T, Kato Y, Hirakata A. Vitrectomy combined with subretinal injection of tissue plasminogen activator for successful treatment of massive subretinal haemorrhage. Eur J Ophthalmol. 2021 Sep;31(5):2588–2595. doi: 10.1177/1120672120970404. Epub 2020 Nov 4. PMID: 33148019.		Excluded	11 eyes
148	Sniatecki JJ, Ho-Yen G, Clarke B, Barbara R, Lash S, Papathomas T, Antonakis S, Gupta B. Treatment of submacular haemorrhage with tissue plasminogen activator and pneumatic displacement in age-related macular degeneration. Eur J Ophthalmol. 2021 Mar;31(2):643–648. doi: 10.1177/1120672119891625. Epub 2019 Dec 9. PMID: 31813290.	75 included (54 eyes)		
149	Tranos P, Tsiropoulos GN, Koronis S, Vakalis A, Asteriadis S, Stavrakas P. Comparison of subretinal versus intravitreal injection of recombinant tissue plasminogen activator with gas for submacular haemorrhage secondary to wet age-related macular degeneration: treatment outcomes and brief literature review. Int Ophthalmol. 2021 Dec;41(12):4037–4046. doi: 10.1007/s10792-021-01976-x. Epub 2021 Jul 30. PMID: 34331185.	76 included (25 eyes)		
150	Fukuda Y, Nakao S, Kohno RI, Ishikawa K, Shimokawa S, Shiose S, Takeda A, Morizane Y, Sonoda KH. Postoperative follow-up of submacular haemorrhage displacement treated with vitrectomy and subretinal injection of tissue plasminogen activator: ultrawide-field fundus autofluorescence imaging in gas-filled eyes. Jpn J Ophthalmol. 2022 May;66(3):264–270. doi: 10.1007/s10384-022-00910-7. Epub 2022 Mar 9. PMID: 35260984.	77 included (24 eyes)		
151	Jackson, T.L., Bunce, C., Desai, R. et al. Vitrectomy, subretinal Tissue plasminogen activator and Intravitreal Gas for submacular haemorrhage secondary to Exudative Age-Related macular degeneration (TIGER): study protocol for a phase 3, pan-European, two-group, non-commercial, active-control, observer-masked, superiority, randomised controlled surgical trial. Trials 23, 99 (2022). https://doi.org/10.1186/s13063-021-05966-3		Excluded	Study protocol
152	Kitagawa Y, Shimada H, Mori R, Tanaka K, Wakatsuki Y, Onoe H, Kaneko H, Machida Y, Nakashizuka H. One-Year Outcome of Intravitreal Tissue Plasminogen Activator, Ranibizumab, and Gas Injections for Submacular Haemorrhage in Polypoidal Choroidal Vasculopathy. J Clin Med. 2022 Apr 13;11(8):2175. doi: 10.3390/jcm11082175. PMID: 35456268; PMCID: PMC9032067.	78 included (64 eyes)		
153	Mehta A, Steel DH, Muldrew A, Peto T, Reeves BC, Evans R, Chakravarthy U; IVAN Study Investigators. Associations and Outcomes of Patients with Submacular Haemorrhage Secondary to Age-related Macular Degeneration in the IVAN Trial. Am J Ophthalmol. 2022 Apr;236:89–98. doi: 10.1016/j.ajo.2021.09.033. Epub 2021 Oct 6. PMID: 34626573.	79 included (535 eyes)		
154	Tiosano A, Gal-Or O, Fradkin M, Elul R, Dotan A, Hadayer A, Brody J, Ehrlich R. Visual acuity outcome in patients with subretinal haemorrhage-office procedure vs. surgical treatment. Eur J Ophthalmol. 2022 May 9:11206721221098208. doi: 10.1177/11206721221098208. Epub ahead of print. PMID: 35532042.	80 included (107 eyes)		
155	Ura S, Miyata M, Ooto S, Yasuhara S, Tamura H, Ueda-Arakawa N, Muraoka Y, Miyake M, Takahashi A, Wakazono T, Uji A, Yamashiro K, Tsujikawa A. Contrast-to-noise ratio is a useful predictor of early displacement of large submacular haemorrhage by intravitreal sf6 gas injection. Retina. 2022 Apr 1;42(4):661–668. doi: 10.1097/IAE.0000000000003360. PMID: 35350046.	81 included (16 eyes)		

## Appendix C

**Author** **(et al.)**	**Year**	**Study Design**	**Study Sample (Eyes)**	**Type of Surgery**	**Mean Size of the Bleeding**	**Outcome Final BCVA**	**Mean Days from Onset**	**Complications**	**GRADE** **^1^**
Olivier [85]	2004	Retrospective, noncomparative, interventional case series	29	PPV + subretinal rt-PA + air	N/A	17 eyes (59%) gained more than 2 lines; 3 eyes (10%) lost more than 2 lines at 3 months	23	2 vitreous hemorrhages and 1 relapse	Very low
Ratanasukon [56]	2005	Retrospective, noncomparative, interventional case series	19	Intravitreal rt-PA + expansile gas	More than 3 disc diameters	BCVA improved 2 lines or greater in 12 eyes (63.2%), stabilized in 6 eyes (31.6%), and worsened in 1 (5.2%) (at 13 months)	13.1	3 vitreous hemorrhages, 3 cataracts, and 2 retinal detachments	Very low
Thompson [90]	2005	Retrospective, comparative, interventional case series	42	PPV + removal of subretinal hemorrhage versus PPV + subretinal rt-PA	12 disc diameters or less	From 20/1000–1 to 20/640–2 at 3 months	N/A	1 RD, 1 hyphema, 2 VH	Low
Yang [58]	2005	Retrospective, comparative, interventional case series	24	Intravitreal injection of expansile gas, with or without adjunctive commercial rt-PA solution	N/A	BCVA improved two or more lines in 11 (45.8%) of the 24 eyes, and measured 20/100 or better in 10 (41.7%) of the 11 eyes	N/A	2 recurrent SRMH, 7 VH, 1 massive VH	Very low
Mozafarieh [27]	2006	Longitudinal prospective study	101	Intravitreous plasminogen activator injection	At least one disc diameter	-	Less than 4 weeks	None	Moderate
Singh [57]	2006	Retrospective, noncomparative, interventional case series	17	PPV + subretinal rt-PA + air	N/A	Improvement was made in all but five patients	Mean duration before surgery was 11.9 days	3 rebleeds, 1 RD	Very low
Chen [58]	2007	Retrospective, noncomparative, interventional case series	85	PPV + subretinal rt-PA + gas	N/A	52 eyes (64%) achieved 2 Snellen acuity lines or better improvement at 3 months	Mean duration of symptoms before surgery was 9.3 days	Vitreous hemorrhage in 8 eyes (8%) and retinal detachment in 3 eyes	Low
Gopalakrishan [28]	2007	Prospective, consecutive, single-center, noncomparative, interventional case series	20	Intravitreal C_3_F_8_ injection without rt-PA	N/A	Mean BCVA improved from 1.6 to 0.72 LogMAR	Range from 1 to 30 days	4 VH	Moderate
Ron [91]	2007	Retrospective, noncomparative, interventional case series	24	PPV + subretinal rt-PA + gas (SF 6 and C_3_F_8_)	At least 3 disc diameters	SF6 used in 13 patients (54.2%). Seven (53.8%) showed improvement of 2 lines on the Snellen chart. C_3_F_8_ was injected in 11 patients (45.8%). Four of them (36.3%) showed an improvement of 2 lines on the Snellen chart	Range from 1 to 30 days	No complications	Low
Stifter [59]	2007	Retrospective, noncomparative, interventional case series	21	Intravitreal bevacizumab	At least 3 disc diameters	VA improved in 48% (10/21) of the treated eyes with a mean VA gain of 0.2 0.16, was stable in 9% (2/21), and decreased in 43% (9/21) of the treated eyes with a mean VA loss of 0.1 0.06	The mean period between symptomatic onset of submacular hemorrhage and the time of initial presentation was 12.9 days	N/A	Very low
Meyer [92]	2008	Retrospective, noncomparative, interventional case series	19	Intravitreal injections of recombined tissue plasminogen activator (rt-PA), expansile gas and bevacizumab	1–3 disc diameters	VA improved two or more lines in 19% of the surgery group compared to 17% of the observation group at 3 months	Onset < 3 months	5 High IOP > 30 mmHg	Very low
Fang [93]	2009	Retrospective, noncomparative, interventional case series	53	PPV with or without subretinal rt-PA + Gas	N/A	Best visual acuity improvement was significantly higher in the rt-PA and gas group than in the gas-alone group (60.7 vs. 32.0%; *p* = 0.037)	Duration < 14 days or > 14 days	VH and endophthalmitis	Low
Arias [60]	2010	Retrospective, noncomparative, interventional case series	15	PPV with intravitreal or subretinal rt-PA + Gas SF6 + intravitreal bevacizumab	1–3 disc diameters	The mean VA (ETDRS) improved from 9.4 letters to 28.2 letters with a mean change of +18.7 letters	Within 5 days from symptoms’ onset	3 VH and 1 RPE tear	Very low
Cakir [61]	2010	Retrospective, noncomparative, interventional case series	21	C_3_ F_8_ gas-assisted pneumatic displacement, with and without intravitreal rt-PA	N/A	Visual acuity in all patients either improved at least one Snellen line (*n* = 13) or remained the same (*n* = 8)	Up to 10 days	2 recurrences	Very low
Fine [94]	2010	Retrospective, noncomparative, interventional case series	15	Subretinal injection of rt-PA (15 of 15) + gas tamponade (12 of 15), oil tamponade (3 of 15)	At least two quadrants	LogMAR 2.77 at baseline vs. 1.95 at 12 months follow-up	Less than 3 weeks after the onset	6 vitreous hemorrhages, 2 RD, 1 glaucoma, 1 cataract, 1 aphakia	Very low
Hillenkamp [35]	2010	Nonrandomized, retrospective, interventional, comparative consecutive series	47	PPV with intravitreal injection of rt-PA and SF6 (Group A) versus PPV with subretinal injection of rt-PA and intravitreal injection of SF6 (Group B)	N/A	The difference in BCVA change between Group A and Group B was not statistically significant	6.6 days (Group A), 5.9 days (Group B)	Group A: 1 vitreous hemorrhage and 1 recurrence; Group B: 3 RD, 2 vitreous hemorrhages	Low
Kung [62]	2010	Retrospective, interventional case series	46	rt-PA + C_3_F_8_	From 0.5 to 35 disc areas (median, 8 disc areas)	Postoperative BCVA improved by 2 Snellen lines or greater in 21 of 45 eyes (46.67%)	10	9 vitreous hemorrhages	Low
Sandhu [63]	2010	Retrospective, interventional case series	16	PPV + submacular rt-PA injection + pneumatic displacement with air followed by postoperative intravitreal ranibizumab (RZB) (12 patients)	6 disc diameters (range 3–12)	At 6 months, 10 of 16 patients had improved by 2 lines; 10 of the 12 patients treated with RZB improved by 2, and all improved by at least 1 line. The mean number of lines of improvement was 3.4	12.5	2 recurrences (treated with another RZB injection)	Very low
Guthoff [36]	2011	Nonrandomized, retrospective, interventional, comparative consecutive series	38	Group A: intravitreal rt-PA + SF6 (26 patients) vs. Group B: intravitreal bevacizumab + rt-PA + SF6 (12 patients)	More than 1 disc diameter	After 7 months, BCVA was significantly higher in the bevacizumab/rt-PA/gas group (B)	1–31 days	No complications	Low
Mizutani [95]	2011	Nonrandomized, retrospective, interventional, comparative consecutive series	53	Intravitreal injection SF6 with/without rt-PA	N/A	Intravitreal SF6 + rt-PA have good visual outcomes and no remarkable complications for treating submacular hemorrhage secondary to AMD. rt-PA is not recommended for ruptured retinal arterial macroaneurysms, because of a higher incidence of subsequent vitreous hemorrhage	N/A	All eyes with macroaneurysms have recurrence. 5 postoperative transient ocular hypertensions	Low
Treumer [64]	2011	Retrospective, consecutive, interventional case series	41	PPV + subretinal rt-PA and bevacizumab + SF6	Mean 4.5 disc diameters (range 1.5–12)	LogMAR BCVA improved significantly from the preoperative value 1.7 (3.0–0.5) to 0.8 (1.6–0.2)	Maximum 2 weeks	8 recurrences	Low
Tsymanava [37]	2012	Retrospective, non-randomized, comparative case study	110	rt-PA injection without gas injection (group A1: 50 µg of rt-PA; A2: 100 µg; A3: 200 µg) and with gas injection (group B1: 50 µg of rt-PA; B2: 100 µg; B3: 200 µg)	12.5 (1–38)	BCVA was better in B1 and B2 groups	10.0 (0.5–180.0)	2 endophthalmitis, 10 vitreous hemorrhages, 3 increased intraocular pressure	Low
Ueda-Arakawa [103]	2012	Nonrandomized, retrospective, comparative consecutive series	31	None, the study valued the retinal structural changes associated with submacular hemorrhage and their relationships with visual prognosis	6.0 ± 3.1 disc areas	The initial OCT detection of the IS/OS just beneath the fovea may predict good visual outcomes	3.7 ± 2.3 months	N/A	Low
Cho [65]	2013	Retrospective, interventional case series	27	Anti-VEGF injection with an initial 3 loading injections by month, followed by an as-needed reinjection in patients with PCV	18.2 ± 13.8 mm^2^	LogMAR visual acuity at baseline was 1.02 ± 0.51 and improved significantly to 0.76 ± 0.48 at 12 months	9.8 ± 7.5 days	3 vitreous hemorrhages	Very low
Han [96]	2013	Retrospective, interventional case series	14	180° peripheral temporal retinotomy, choroidal neovascular membrane (CNVM) excision, and transplantation of an autologous simple RPE sheet developed from the PED region outside the CNVM lesion	Massive submacular hemorrhage (extending to at least one temporal vessel arch)	Mean ETDRS score increased from 14.0 ± 23.4 preoperatively to 31.9 ± 23.8 at 18 months	Less than 6 months	1 RD, 1 delayed recurrent submacular hemorrhage	Very low
Jain [66]	2013	Retrospective chart review	14	Anti-VEGF monotherapy	More than 2 disc areas	Mean change in VA from baseline at final follow-up was −0.58 LogMAR (range −1.6 to +1, Snellen range 20/30–20/400, median 20/60; *p* = 0.0022)	4 (range 1–7)	N/A	Very low
Mayer [38]	2013	Nonrandomized, retrospective, interventional, comparative consecutive series	45	Group A: rt-PA (50 µg⁄0.05 mL) + SF6. Group B: bevacizumab (1.25 mg⁄0.05 mL) + SF6. Thereafter, all patients received bevacizumab	From 1 to 5 disc areas	Better VA in the rt-PA and gas group (14 letters improvement) compared with VA increase in the bevacizumab and gas group (8 letters improvement) from baseline to 12 months follow-up (*p* < 0.03)	N/A	10 vitreous hemorrhages	Low
Rishi [39]	2012	Retrospective, single-center study	46	PPV + subretinal rt-PA (group 1); pneumatic displacement with intravitreal rt-PA and gas (group 2); pneumatic displacement with intraocular gas (group 3)	5.6 ± 3.4 disc areas	No statistically significant difference amongst groups	10	4 cataracts, 4 RD, 2 secondary glaucoma, 2 vitreous hemorrhages	Low
Shienbaum [97]	2013	Retrospective, interventional, consecutive case series	19	Anti-VEGF monotherapy	39.0 mm^2^ (range 4.3–170.2 mm^2^	The mean change in approximate ETDRS letter score from baseline was +12 letters at 3 months (*p* < 0.003), +18 letters at 6 months (*p* < 0.001), and +17 letters at 12 months follow-up (*p* < 0.02)	N/A	No adverse ocular or systemic events	Very low
Chang [98]	2014	Retrospective, comparative, interventional case series	101	PPV + subretinal rt-PA + gas tamponade with and without postsurgical antiVEGF injection	N/A	BCVA of the group that received post-operative anti-VEGF injection showed greater visual acuity improvement 6 months postoperatively compared to the group that did not receive post-operative anti-VEGF	N/A	6 recurrences, 4 RD, 2 vitreous hemorrhages	Low
Iacono [29]	2014	Prospective interventional case series	23	Intravitreal ranibizumab	Occult choroidal neovascularization with flat large submacular hemorrhage > 50% of the entire lesion	At 12-months mean visual acuity improved significantly to 0.68 ± 0.41 (*p* = 0.04)	N/A	None	Very low
Kitahashi [40]	2014	Retrospective, comparative, interventional case series	32	Pneumatic displacement (SF6) + intravitreal bevacizumab vs. pneumatic displacement (SF6) alone	More than 2 disc areas	Mean BCVA in the SF6 + IVB group was significantly better than that in the SF6 group at 1, 3, and 6 months postoperatively (*p* 0.001, *p* 0.001 and *p* 0.001, respectively)	Less than 10 days	1 RD	Low
Moisseiev [67]	2014	Retrospective, noncomparative, interventional case series	31	PPV + subretinal rt-PA	N/A	Patients who improved by at least one line in VA (change in LogMAR −0.41 ± 0.17) had worse VA at presentation and better final VA than those who did not improve (change in LogMAR 0.37 ± 0.18)	13.4 ± 12.4 (range 1–60) days	6 RD, 2 recurrences, 2 elevated IOP following surgery	Low
Dimopoulos [41]	2015	Retrospective, noncomparative, interventional case series	46	Intravitreal bevacizumab in group A (from 1 to 4 disc area), group B (from 4 to 9 DA), group C (more than 9 DA)	6 DA	The mean BCVA increased from 0.81 LogMAR (Snellen 20/125) at baseline to 0.75 LogMAR (20/125) after 1 year. Amongst the three groups, improvement of the BCVA was found in 57% (13/23), 53% (8/15), and 38% (3/8) of eyes, respectively.	11.5 ± 19 days (range: 1–45 days)	None	Low
Kadonosono [86]	2015	Prospective, consecutive, interventional case series	13	25-gauge vitrectomy and submacular injection of rt-PA and air	N/A	Mean ETDRS score improvement was 19.4 letters at 1 month and 23.3 letters at 3 months	19 days (range 7–40)	1 FTMH, 1 VH	Moderate
Kim [68]	2015	Retrospective, noncomparative, interventional case series	49	Intravitreal anti-VEGF injection	13.9 ± 8.8 disc areas	Mean BCVA improved from 1.14 ± 0.61 LogMAR (20/276, Snellen equivalent) to 0.82 ± 0.53 LogMAR (20/132) at 12 months	7.25 ± 5.9 days	10 VH	Low
Kimura [69]	2015	Prospective, noncomparative, interventional case series	15	PPV + subretinal rt-PA injection + air tamponade, followed by intravitreal antiVEGF injection	5.6 ± 4.7 disc diameters (range, 1.5–20 disc diameters)	Mean BCVA at baseline (0.98 ± 0.44) had improved significantly both 1 month after surgery (0.41 ± 0.25) and at final visit (0.23 ± 0.25)	9.5 ± 4.5 (range 5–21) days	None	Moderate
Shin [42]	2015	Retrospective, comparative, interventional case series	82	Pneumatic displacement (SF6 or C_3_F_8_) + intravitreal anti-VEGF vs. anti-VEGF monotherapy	N/A	Improvement in BCVA was not significantly different between the 2 groups at baseline, 3 months, or 6 months after initial treatment, but the combination therapy group showed better visual acuity at 1 month after initial treatment compared with the monotherapy group	11.4 ± 10.4 days in the combination therapy group and 13.8 ± 11.5 days in the monotherapy group	6 recurrences, 12 RPE tears, 5 VH, 2 RRD, 1 HRD with choroidal hemorrhage	Low
Wei [30]	2015	Prospective, nonrandomized, and noncomparative case series study	21	PPV + 360° retinotomy + silicon oil (Oxane 5700) tamponade	N/A	The mean BCVA in LogMAR (Snellen equivalent) significantly improved from preoperatively 2.64 (hand movement) to 1.73 (7/400), 1.50 (6/200), 1.51 (6/200), and 1.45 (7/200) at 1 month, 3 months, 6 months after the initial surgery, and final follow-up	N/A	10 mild subretinal fibrosis	Moderate
Bae [105]	2016	Retrospective, interventional case series	37	Pneumatic displacement + laser or anti-VEGF	12.9 ± 8.3 DA	The mean BCVA was 1.08 ± 0.55 at baseline, 0.74 ± 0.57 at 3 months, and 0.63 ± 0.58 at 6 months. The mean BCVA of PCV patients was significantly better than that of typical exudative AMD patients	16.1 ± 12.5	None	Low
De Jong [31]	2016	Prospective, noncomparative, interventional case series	24	Group A: PPV + gas + subretinal rt-PA; Group B: intravitreal rt-PA + gas	Group A: 11.1 DA (range 0.5–31.0); Group B: 9.7 DA (range 2.9–20.2)	Median visual acuity improvement in ETDRS lines at Week 4, 6, and 12 shows no significant differences between groups	Group A: 5 (range 1–11), Group B: 6 (range 1–14	Group A: 3 increased IOP > 50 mmHg, 2 VH, 1 RD, 1 recurrence. Group B: 2 RD, 2 recurrences	Very low
Fassbender [43]	2016	Retrospective case series	39	Group A: PPV + subretinal (rt-PA) injection; Group B: pneumatic displacement (PD) + intravitreal rt-PA; Group C: PD without rt-PA	Group A: 9.1 mm^2^; Group B: 8.1 mm^2^; Group C: 9.1 mm^2^	Final visual acuity improved significantly in both the vitrectomy and subretinal rt-PA injection group and the intravitreal rt-PA injection group, but not with PD alone	Group A: 5 ± 4.6; Group B: 6 ± 4.2; Group C: 6 ± 2.2	None	Low
Gonzàlez-Lòpez [70]	2016	Retrospective, nonrandomized, and noncomparative case series study	45	Small gauge PPV + subretinal rt-PA + ranibizumab	40.64 ± 20.20 mm^2^	Visual acuity improved −0.59 ± 0.61 LogMAR between presentation and last follow-up	6.98 ± 5.70	N/A	Low
Kitagawa [32]	2016	Prospective, interventional, consecutive case series	20	Intravitreal rt-PA + ranibizumab + gas without vitrectomy	11.1 ± 8.7 disc diameters (range, 2–31 disc diameters)	Mean change in ETDRS score from baseline was +13 letters	9.9 ± 9.8 days (range 2–30 days)	3 VH, 1 RD	Very low
Lee [71]	2016	Retrospective clinical case series	25	Intravitreal injections of tenecteplase, antiVEGF, and expansile gas	7.5 ± 5.0 disc areas (range, 1.5 to 19)	BCVA improved from 1.09 ± 0.77 LogMAR at baseline to 0.52 ± 0.60 LogMAR at 12 months	7.2 ± 8.2 days (range, 1 to 30 days)	1 VH, 1 RPE tear	Very low
Lin [44]	2016	Retrospective, comparative, interventional case series	20	Group A: subretinal rt-PA + PPV; Group B: or intravitreal rt-PA + gas to achieve pneumatic displacement. Additionally, combination treatment with either (PDT) or intravitreal injection of anti-VEGF was performed	17.8 ± 19.2 disc diameter (DD) compared (2.64 DD)	Combination treatment with PDT showed significant efficacy in the improvement of BCVA	14.3 ± 16.6 days	2 VH	Very low
Waizel [72]	2016	Observational analysis	83	PPV + rt-PA + gas for subretinal hemorrhages (SRH), subpigment epithelial hemorrhages (SPH), and combined subretinal and subpigment epithelial hemorrhages (CH). 68.7% received additional anti-VEGF	N/A	Vitrectomy combined with subretinal rt-PA injection and gas or air tamponade has a strong functional and anatomical effect on both SRH and CH and also seems to slightly improve the anatomical outcome in SPH	13.4 ± 13.4 in CH; 8.3 ± 10.3 in SRH; 4.9 ± 8.0 in SPH	1 FTMH, 1 RD, 3 recurrences	Low
Bell [45]	2017	Retrospective chart review	18	Pneumatic displacement followed by intravitreal rt-PA if needed vs. PPV with subretinal rt-PA	N/A	The percentage of patients achieving three lines or greater improvement at 1 year was 46% and 18% in these groups; the difference was not statistically significant	N/A	1 recurrence	Very low
Gok [73]	2017	Retrospective, nonrandomized, consecutive case series	17	PPV + subretinal rt-PA + SF6	8.6 ± 5.3 disc areas	Improvement from initial VA (mean LogMAR, 1.8 ± 0.3) and the final BCVA (mean LogMAR, 0.97 ± 0.52)	12.8 ± 18.2 days	1 recurrence, 1 RRD	Very low
Waizel [74]	2017	Retrospective observational study	85	PPV with rt-PA combined with gas or air tamponade	N/A	In air tamponade group, mean VA improved from LogMAR 1.42 ± 0.52 to LogMAR 1.25 ± 0.51 (20/530 to 20/355 Snellen equivalent, average gain in visual acuity of 1.7 lines). In gas tamponade group, VA improved from LogMAR 1.37 ± 0.42 to LogMAR 1.29 ± 0.39 (20/471 to 20/394 Snellen equivalent, average gain in visual acuity of 0.8 lines)	13.5 ± 12.3 days in gas tamponade; 10.9 ± 15.2 days in air tamponade	1 FTMH, 3 recurrences	Low
Bardak [75]	2018	Retrospective pilot study	16	Intravitreal rt-PA + pneumatic displacement + anti-VEGF	N/A	Mean BCVA was 2.08 ± 0.79 LogMAR at baseline, 1.09 ± 0.73 LogMAR at the last follow up	7.9 ± 3.6 days	1 retinal pigment epithelium tear	Very low
Juncal [84]	2018	Retrospective case series	99	PPV + subretinal rt-PA + pneumatic displacement	15.7% 1–5 DA; 18.6% 6–10 DA; 65.7% > 10 DA	Mean LogMAR BCVA improved from 2.03 ± 0.81 (Snellen 20/2143) at baseline to 1.80 ± 1.00 (Snellen 20/1262) at final follow-up	19.6 ± 29.1 (2–160)	13 VH, 8 RD, 4 RPE tears, 2 expulsive choroidal hemorrhages, 12 recurrences	Low
Kim [87]	2017	Retrospective observational study	21	Anti-VEGF monotherapy	19.3 ± 9.1 mm^2^ (range: 5.6 to 32.3 mm^2^)	From 0.86 ± 0.39 LogMAR at baseline to 0.53 ± 0.43 LogMAR after resolution of the hemorrhage	16.0 ± 10.1	N/A	Very low
Kimura [99]	2018	Retrospective analysis	33	Pneumatic displacement with SF6 with or without rt-PA	N/A	The BCVAs improved significantly in eyes with PCV compared with eyes with typical AMD	N/A	4 persistent PED	Low
Sharma [76]	2017	Retrospective, noncomparative, interventional case series	24	PPV + subretinal air injection in combination with rt-PA + partial fluid–gas exchange and preoperative, intraoperative, or postoperative anti-VEGF	Small (does not reach arcades) in 6 patients (25%), large (extending to the arcades) in 2 patients (8.3%), extensive (extending past the arcades) in 9 patients (37.5%), and massive (extending to 2 quadrants, past the equator, or both) in 7 patients (29.2%)	Mean preoperative VA was 1.95 (Snellen equivalent, 20/1783), mean postoperative VA was 0.85 LogMAR (Snellen equivalent, 20/141)	11.3 days (range, 1–59 days; median, 9 days)	5 recurrences, 3 VH, 2 RD, 1 FTMH	Very low
Plemel [100]	2019	Retrospective, noncomparative, interventional case series	78	PPV + subretinal rt-PA + gas tamponade	13.6 DA	Visual acuity improved from 20/1449 preoperatively to 20/390 after a mean follow-up time of 6.3 months, corresponding to approximately 5 lines of Snellen acuity improvement	N/A	9 recurrences	Low
Helaiwa [101]	2020	Single-center, case series report	14	Intravitreal rt-PA + (next day) PPV + subretinal rt-PA + air tamponade	21.6 ± 17.8 mm^2^	Significant (*p* = 0.01) overall improvement in the visual acuity post-treatment (from 1.4 ± 0.5 log MAR to 0.9 ± 0.4)	N/A	None	Very low
Jeong [46]	2020	Retrospective chart review	77	Group A: anti-VEGF monotherapy, Group B: PD + anti-VEGF; Group C: PPV + subretinal rt-PA and gas tamponade	Three groups according to the dimensions: small-sized (optic disc diameter (ODD) ≥1 to <4), medium-sized (ODD ≥ 4 within the temporal arcade) and large-sized (ODD ≥ 4, exceeding the temporal arcade)	In the small-sized group, all treatment modalities showed a gradual BCVA improvement. In the medium-sized group, PD and surgery were associated with better BCVA than anti-VEGF monotherapy (67% and 83%, respectively, vs. 33%). In the large-sized group, surgery showed a better visual improvement with a higher displacement rate than PD (86% vs. 25%)	14.3 ± 25.8	1 recurrence, 1 RPE rip, 1 FTMH	Low
Karamitsos [77]	2020	Retrospective case series	28	Intravitreal rt-PA + gas ± anti-VEGF	4.9 mm in patients with no anatomical displacement; 4.5 ± 1.4 mm in patients with anatomical displacement	The mean improvement of all patients with anatomical displacement of the hemorrhage in visual acuity was 0.7 ± 0.5 (LogMAR) in 1 month	3.5 ± 2.1 days in patients with no anatomical displacement and 3.4 ± 3.6 days in patients with anatomical displacement	2 VH, 1 RD	Very low
Kim [104]	2019	Retrospective, nonrandomized, and noncomparative case series study	29	Intravitreal aflibercept	6.2 ± 4.8 DA	BCVA significantly improved from 52.9 ± 17.8 ETDRS letters at week 0 to 71.8 ± 16.1 letters at week 56	≤7 days in 18 patients (62.1%), >7 days but ≤1 month in 7 patients (24.1%), and >1 month but <3 months in 4 patients (13.8%)	8 reactivations	Very low
Lee [78]	2020	Retrospective, comparative, interventional case series	67	Pneumatic displacement among patients with different subtypes of age-related macular degeneration (AMD): typical AMD, polypoidal choroidal vasculopathy (PCV), and retinal angiomatous proliferation (RAP)	6.63 ± 4.66 DA in typical AMD; 5.95 ± 4.58 DA in PCV; 8.35 ± 4.00 DA in RAP	The proportion of eyes with improved visual acuity was highest in the PCV subtype and lowest in the RAP subtype	7.85 ± 6.89	N/A	Low
Maggio [47]	2020	Retrospective, noncomparative, interventional case series	96	Intravitreal rt-PA + SF6 for guiding the selection of additional treatments (anti-VEGF, PDT, or submacular surgery) or observation (CNV)	At least 3 DA involving the fovea	BCVA improved from 1.8 (SD = 0.96) LogMAR to BCVA significantly improved to 1.29 (SD = 0.78) LogMAR at 3 months	Less than 14 days	7 recurrences, 2 VH	Low
Wilkins [102]	2020	Retrospective, interventional case series	37	PPV + subretinal rt-PA + pneumatic displacement	N/A	Median preoperative VA was 20/2000, at postoperative month 3 was 20/152	N/A	5 VH, 4 recurrent H, 3 RD, 1 blood stained cornea, 1 glaucoma, 1 phthisis	Low
Ali Said [79]	2021	Retrospective analysis	93	PPV + subretinal rt-PA + air	N/A	Mean BCVA at baseline was 0.06 Snellen; after the surgery, BCVA improved to 0.16 Snellen; at 8 months, decreased to 0.08 Snellen	The majority of eyes were operated within two days after the onset of the hemorrhage (60, 2%), 90.3% of eyes within one week, and all 93 eyes within 14 days	2 RPE tears, 7 VH, 4 hyphema, 6 RD, 2 subchoroidal hemorrhages	Low
Avci [80]	2021	Retrospective study	30	PPV + C_3_F_8_ 5% + subretinal rt-PA + anti-VEGF	61.95 ± 43.47 (range: 10.75–176.42) mm^2^	Mean VA improved from LogMAR 2.11 ± 0.84 at baseline to LogMAR 1.32 ± 0.91, 0.94 ± 0.66, 1.13 ± 0.84, and 1.00 ± 0.70 at postoperative month 1, 2, 3, and 6, respectively	13.70 ± 8.05 (range: 2–30) days	2 recurrences	Low
Caporossi [88]	2022	Retrospective, consecutive, comparative, non-randomized interventional study	44	Group A: PPV + massive submacular hemorrhage and neovascular membrane removal + hAM subretinal implant + silicone oil; Group B: PPV + subretinal rt-PA + SF6 20%	N/A	Mean preoperative BCVA was 1.9 LogMARin the amniotic membrane group and 2 LogMAR in the rt-PA group. The mean final BCVA values were 1.25 and 1.4 LogMAR, respectively, with a statistically significant difference	25.9 ± 36 days (range, 1–150 days)	2 VH, 2 RD	Low
Iannetta [81]	2021	Single-center, retrospective, case series	25	PPV + subretinal rt-PA + SF6 20%	68.34 ± 42.42 mm^2^	BCVA significantly improved from 1.81 ± 0.33 to 1.37 ± 0.52 LogMAR at 12 months from surgery	9.24 (±3.37)	2 recurrences, 1 RD	Very low
Asli [48]		Retrospective case series	54	- 21 eyes PPV + submacular rt-PA + 20% SF6 or 14% C_3_F_8_; - 14 eyes PPV + submacular rt-PA + 20% SF6 or 14% C_3_F_8_ + antiVEGF; - 10 eyes PPV + subretinal rt-PA without gas; - 4 eyes intravitreal gas + rt-PA; - 3 eyes PPV + subretinal rt-PA + drainage; - 2 eyes intravitreal gas + intravitreal antiVEGF	31.5 ± 26.5 (2.8–145.3) mm^2^	BCVA improved from 2.0 ± 0.8 (0.3–3) at presentation to 1.67 ± 0.87 (0.22–3) LogMAR on postoperative month 1, 1.65 ± 0.79 (0.40–3) LogMAR on month 3, 1.76 ± 0.88 (0.22–3) LogMAR on month 6, 1.71 ± 0.85 (0.30–3) LogMAR at year 1, and 1.76 ± 0.81 (0.22–3) LogMAR at final visit	13.7 ± 16.3 (1–95) days	6 RD, 3 VH, 2 FTMH, 4 recurrences	Low
Kawakami [82]	2021	Retrospective case series	25	PPV ± anti-VEGF (2 eyes in SMH group)	4.0 ± 1.6 (range 2.0–6.7) disc diameters	BCVA improved in eyes with SMH at 6 and 12 months after PPV, and the BCVA was maintained until the end of the study (24 months)	9.3 ± 4.6 (range 4–17)	2 FTMH	Very low
Kishikova [49]	2020	Retrospective analysis	29	Group 1: intravitreal rt-PA + SF6 Group 2: PPV + subretinal rt-PA + SF6 with (2A) or without (2B) subretinal air	9.45 DD ± 2.34 in Group 1 and 9.72 DD ± 2.02 in Group 2	The mean BCVA at presentation was 0.0068 in Group 1 and 0.0067 in Group 2. The mean postoperative BCVA at 6 months was 0.31 in Group 1 and 0.58 in Group 2. Subgroup analysis of Group 2 did not show statistically significant difference in outcome when adding subretinal air to the vitrectomy procedure	N/A	3 high IOP, 2 RD, 3 cataracts	Very low
Pierre [13]	2021	Multicentric retrospective case series	65	PPV + subretinal rt-PA + gas	5441.3 ± 1323.1 microns in AMD group; 5085.2 ± 3012.3 microns in RAM group	BCVA improved from 20/500 to 20/125 at month 1 and month 6	7.1 ± 6.7 days (range 0–30 days)	7 VH, 5 RRD, 7 recurrences	Low
Matsuo [50]	2021	Retrospective study	30	13 eyes underwent pneumatic displacement, 22 eyes received anti-VEGF therapy, and four eyes underwent PPV	17.0 ± 4.8 disc areas	The mean BCVA at the development, 1 month, and 1 year after the development of large SMHs, and at the final visit were LogMAR 0.88 ± 0.61 (Snellen equivalent 20/151), LogMAR 1.12 ± 0.82 (Snellen equivalent 20/263), LogMAR 0.84 ± 0.72 (Snellen equivalent 20/138), and LogMAR 0.88 ± 0.75 (Snellen equivalent 20/152), respectively	N/A	2 VH, 1 RRD	Low
Rickmann [51]	2021	Retrospective, consecutive case series	31	PPV + subretinal rt-PA + air displacement with (group +B) or without Group − B) intravitreal bevacizumab	11.78 ± 3.04 mm^2^ in +B; 14.75 ± 3.98 mm^2^ in −B	The mean visual acuity improved significantly in both groups, from 1.37 ± 0.39 to 1.03 ± 0.57 LogMAR in +B and from 1.48 ± 0.48 to 1.01 ± 0.38 LogMAR in group B	3.3 ± 1.6 in +B; 3.4 ± 1.5 in −B	1 FTMH, 2 RD, 1 recurrence	Low
Sniatecki [52]	2019	Retrospective study	54	PPV + subretinal rt-PA + pneumatic displacement vs. antiVEGF monotherapy	In rt-PA group, 5.0 DA; in anti-VEGF group, 4.2 DA	Mean LogMAR in rt-PA group at baseline was 1.56, and it was improved at 1 year to 1.07. Mean BCVA in anti-VEGF group at baseline was 1.22 LogMAR with no significant improvement (1.36 at 1 year)	5 days (range 1–13)	2 RD, 3 VH, 7 cataracts	Low
Tranos [53]	2021	Retrospective, comparative, interventional case series	25	Group A: PPV + subretinal rt-PA + gas; Group B: intravitreal rt-PA + gas	4.604 ± 2079 µm	BCVA improved significantly but did not differ between the 2 groups	8.2 (±7.3) days	N/A	Very low
Fukuda [83]	2022	Retrospective, consecutive case series	24	PPV + subretinal rt-PA	N/A	BCVAs at baseline (1.16 ± 0.52) had improved significantly at the final visit (0.52 ± 0.39)	11.5 ± 6.6 days	N/A	Very low
Kitagawa [33]	2022	Extended study of a previous prospective study	64	Intravitreal rt-PA + ranibizumab + gas injection	8 ± 6 (range, 2–27) disc diameters	Mean ETDRS score increased from 58 at baseline to 64 letters	7 ± 7 (range, 1–30) days	46 recurrences	Low
Mehta [34]	2021	Secondary analyses of a randomized, controlled trial of image and clinical data	535	Randomly divided into monthly ranibizumab, as-needed ranibizumab, monthly bevacizumab or as-needed bevacizumab	89% were smaller than 1 standard disc area	SMH at baseline was associated with worse baseline BCVA compared with eyes with no SMH (median letters, 62 vs. 68; *p* < 0.001; estimate of difference 6 letters, 95% CI, 4–8 letters). By month 12, the BCVA had improved in both groups (median letters 71 vs. 75) and was not significantly associated with the presence of baseline SMH (*p* = 0.570)	N/A	1 RPE tear, 28 fibrosis, 10 atrophic scars, 6 geographic atrophies, 7 epiretinal membranes	Moderate
Tiosano [54]	2022	Retrospective study	107	Group A: intravitreal injection of rt-PA + gas; Group B: PPV	767 microns in Group A and 962.5 microns in Group B	Visual acuity (in LogMAR) was similar in the two groups prior to the diagnosis of subretinal hemorrhage but better in the rt-PA and gas group at the end of follow-up	N/A	N/A	Low
Ura [89]	2022	Retrospective study	16	Investigate predictors of early displacement of submacular hemorrhage by simple intravitreal SF6 gas injections before inception of subretinal rt-PA	33.10 ± 13.98 mm^2^	LogMAR BCVA at 1 week after the injection showed no statistically significant association with any of the measured parameters	20.6 ± 44.2 days	N/A	Very low
^1^ Grading of Recommendations Assessment, Development and Evaluation (GRADE) system [25,26].									
